# Polyoxygenated Stigmastane-Type Steroids from *Vernonia kotschyana* Sch. Bip. ex Walp. and Their Chemophenetic Significance †

**DOI:** 10.3390/molecules28135278

**Published:** 2023-07-07

**Authors:** Nadine Tseme Wandji, Gabin Thierry M. Bitchagno, Isabelle Mawabo Kamga, Joseph Tchamgoue, Celine Nguefeu Nkenfou, Bruno Ndjakou Lenta, Norbert Sewald, Simeon Fogue Kouam

**Affiliations:** 1 Department of Chemistry, Higher Teacher Training College, University of Yaounde I, Yaounde P.O. Box 47, Cameroon; nadtseme@yahoo.fr (N.T.W.); tchamgouej@yahoo.fr (J.T.); lentabruno@yahoo.fr (B.N.L.); 2 Department of Organic Chemistry, Faculty of Science, University of Yaounde I, Yaounde P.O. Box 812, Cameroon; 3 Organic and Bioorganic Chemistry, Faculty of Chemistry, Bielefeld University, D-33501 Bielefeld, Germany; gabin1256@gmail.com; 4 Department of Chemistry, University of Dschang, Dschang P.O. Box 67, Cameroon; 5 Department of Biology, Higher Teacher Training College, University of Yaounde I, Yaounde P.O. Box 47, Cameroon; bibiisabelle@yahoo.fr (I.M.K.); nkenfou@yahoo.com (C.N.N.); 6 Molecular Biology Center, Yaounde P.O. Box 14495, Cameroon

**Keywords:** *Vernonia kotschyana*, Asteraceae, stigmastanes, structure elucidation, chemophenetic significance, antibacterial activity

## Abstract

Four polyoxygenated stigmastanes (**1**–**4**) alongside known analogues (**7**–**8**) and flavonoids (**5**–**6**) were isolated from a dichloromethane/methanol (1:1, *v*/*v*) extract of the whole plant of *Vernonia kotschyana* Sch. Bip. ex Walp. (Asteraceae). Their structures were determined by means of spectroscopic and spectrometric analysis. The relative stereochemistry of the new compounds was established and confirmed via biosynthesis evidence and cyclization of **1** under acidic conditions. A plausible biosynthetic pathway to the new compounds and the chemophenetic significance of the isolated constituents were also discussed. The crude extract, fractions, and compounds (**1**–**3**) were assessed for their antibacterial activity against five highly prevalent bacterial strains. The fractions and compounds showed low to moderate activity with minimal inhibitory concentrations (MICs) > 125 µg/mL.

## 1. Introduction

The genus *Vernonia* contributes to alleviating malaria and other parasitic illnesses in tropical regions Worldwide [1,2,3]. The species *V. amygdalina* (locally called “Ndolès” in Cameroon) was the first within the genus to arouse medicinal interests following the uses of plant leaves by wild chimpanzees when experiencing fever [2,4]. Ever since, extensive investigations have reported on the occurrences of sesquiterpenoid lactones and stigmastane-type steroids as chemical markers of the genus [3,4,5,6]. Unlike sesquiterpene lactones, which are distributed throughout the genus, stigmastanes instead occur in few species, including *V. guineensis* and *V. amygdalina* [4,5,6,7,8]. Sesquiterpene lactones are able to alkylate cysteine-containing enzymes and proteins, inducing apoptosis in malignant cells [9,10]. Sesquiterpene lactones and related enriched extracts have shown significant potential against various cancer cells [11,12].

The genus *Vernonia* constitutes a group of 350 species that include *Vernonia kotschyana* Sch. Bip. Ex Walp, which is the accepted name in the genus of *V. adoensis* var. kotschyana (Sch.Bip. ex Walp.) G. V. Pope, *V. bequaertii* De Wild., *V. kotschyana* Sch. Bip. ex Walp., *V. kotschyi* Sch. Bip. ex Schweinf., *V. leptolepis* Bak., and *V. woodii* O. Hoffm. It is distributed around tropical regions in Africa from Senegal to Cameroon and extending across the continent to Ethiopia [11,12]. It is an herbal remedy used in African folk medicine against digestive insufficiency, colitis, dermatosis, tuberculosis, and headache. The roots of the plant are used in Mali for the treatment of gastritis, stomach ulcer, and wounds. Traditional healers recommend the use of the decoction of dried and powdered roots in the treatment of gastric dysfunction [13]. It was even considered for clinical trials in Mali and has demonstrated efficacy in alleviating gastric ulcers in patients; it therefore has been listed amongst the essential drugs of the country by the Ministry of Health since 2005 [12,14,15]. In the Southwest Region of Cameroon (Lebialem Division), the maceration of leaves in water as well as the decoction of roots of the plant are taken orally for the treatment of gastritis and internal ulcers [16]. A recent study of the total metabolome of *V. kotschyana* by means of LC-HR-ESI-MS/MS revealed the presence of highly oxygenated stigmastane-type saponins in the roots, including vernocuminosides I-J; vernoamyoside D; vernoniosides D1, D2-D3, and F1-F2; and vernoniamyoside A-D [17] in accordance with previous findings by Sanago et al. 1997 [18], Nergard et al. also revealed the occurrences of pectic arabinogalactan type II in the roots [12,14,15]. Biological studies acknowledge the involvement of the plant saponins and polysaccharides in the immunomodulating inhibition of *Helicobacter pylori*, cancer cell viability, apoptosis, and ROS production properties of the plant roots [14,15,16,17,19]. Within the frame of the ongoing research project on the search for taxa with antibacterial and antiparasitic activities from Cameroonian rain forests and pharmacopeia [20,21,22], we undertook a phytochemical investigation and antibacterial screening of the whole plant of *V. kotschyana*. Four hitherto unknown stigmastane-type steroids (**1**–**4**) alongside the antibacterial activities of the extract, fractions, and compounds are herein reported.

## 2. Results and Discussion

**Figure 1:**
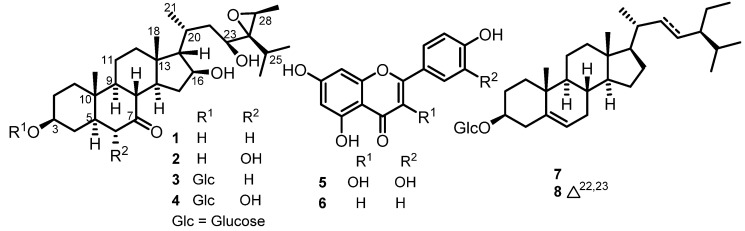
Structures of isolated compounds **1**–**8** from *V. kotschyana*.

The DCM/MeOH (1:1) extract of the whole plant of *V. kotschyana* was subjected to silica gel flash column chromatography, yielding four main fractions that were further purified via various chromatographic methods to afford eight secondary metabolites, including four new stigmastane-type steroids (**1**–**4**) (Figure 1). Their structures were determined by means of extensive spectroscopic and spectrometric analysis.

### 2.1. Structure Elucidation of Compounds

**Figure 2:**
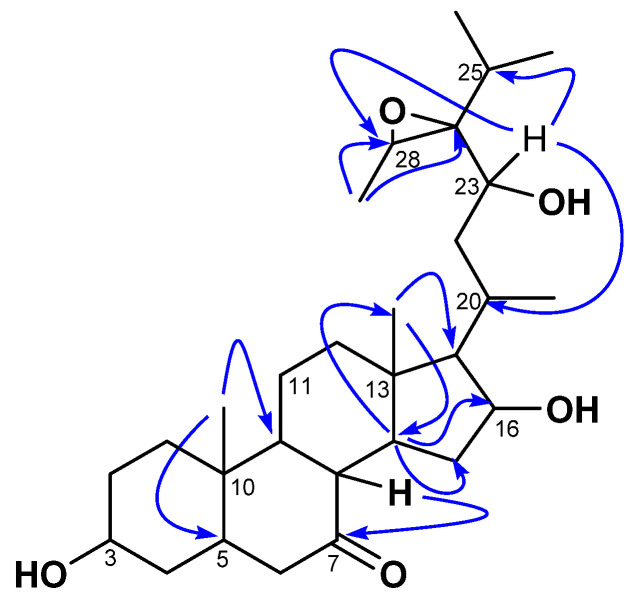
Key HMBC correlations of kotschyanoside A (**1**).

Compound **1** was isolated as a white amorphous powder with an ${\left[\alpha \right]}_{D}^{20}$ of −23 (*c* 0.5, MeOH). Its molecular formula (C_29_H_48_O_5_) was determined via HR-ESI-MS, which showed the sodium adduct ion peak [M+Na]^+^ at *m/z* 499.3386 (calcd. for C_29_H_48_O_5_Na^+^, 499.3399). Its IR spectrum showed characteristic absorption bands of hydroxyl (3392 cm^−1^), carbonyl (1735 cm^−1^), and aliphatic chains (2940 and 2870 cm^−1^). The ^1^H NMR spectrum of **1** was typical of a sterol structure, displaying two angular methyl singlets of stigmastane-type steroids at *δ*_H_ 1.06 (3H, s, H-18) and 1.09 (3H, s, H-19) assigned based on the HMBC interaction from the signal at *δ*_H_ 1.06 to the characteristic carbon C-5 (*δ*_C_ 47.5) of triterpenes [23,24,25]. The stigmastane backbone was confirmed by the ^13^C NMR spectrum of **1,** which revealed the occurrences of 29 carbon atoms (including a ketonic carbonyl group at *δ*_C_ 212.2 (C-7). However, the spectrum showed signals of more than one oxygenated carbon (60–85 ppm) not common in steroids. Moreover, the ^13^C NMR spectrum also lacked C-C double bond signals to decide on the type of stigmastane skeleton, stigmasterol, or sitosterol; while the ^1^H NMR spectrum showed more than one signal for methyl doublets. Nevertheless, the HMBC spectrum of **1** evidenced scalar coupling from the methyl H-19 (*δ*_H_ 1.09) to the methine C-9 (*δ*_C_ 55.9), which was part of a spin system formed by H-8/H-9/H-14 as judged by cross-peaks between signals at *δ*_H_ 2.41, 1.08, and 1.47 from the ^1^H-^1^H COSY spectrum. Thus, interactions of the latter—mainly from H-9/H-14—helped to locate the carbonyl at C-7. Further interactions from H-14 to C-13 (*δ*_C_ 43.1), C-15 (*δ*_C_ 37.4), and C-16 (*δ*_C_ 72.3) evidenced an oxygenated methine at C-16. The proton H-16 of the latter was part of a spin system along with H-15 (*δ*_H_ 3.26) and H-17 (*δ*_H_ 0.96, m) as judged by correlations between H-15/H-16/H-17 observed in the ^1^H-^1^H COSY spectrum. One of the secondary methyls at *δ*_H_ 1.03 and the angular methyl singlet H-18 (*δ*_H_ 1.06) further showed HMBC cross-peaks to C-17 at *δ*_H_ 62.0. The position of the oxygenated methine CH-23 was determined based on ^1^H-^1^H COSY correlations between the doublet at *δ*_H_ 1.03 (ultimately assigned to H-21), and the methine H-20 (*δ*_H_ 2.47), then between H-20 and H-17 (*δ*_H_ 0.96) and the methylene H-22 (*δ*_H_ 1.45), and finally between H-22 to H-23 (*δ*_H_ 4.28). The other secondary methyls at *δ*_H_ 1.10 (3H, d, *J* = 7.0 Hz) and 1.14 (3H, d, *J* = 7.2 Hz) were part of a spin system with the methine at *δ*_H_ 1.82 (H-25) characteristic of the isopropyl moiety, which is common in stigmastanes [25]. The remaining secondary methyl at *δ_H_* 14.2 (assigned to H-29) showed HMBC cross-peaks (Figure 2) to two oxygenated methine carbons at *δ_C_* 69.3 (C-24) and 57.2 (H-28). The relative low resonances of both C-28 and C-24 combined with the molar mass and hydrogen bond deficiency imposed an epoxide between C-24 and C-28 [25,26]. Consequently, the planar structure of **1** was deduced to be 24,28-epoxy-3,6,23-trihydroxystigmastan-7-one.

**Figure 3:**
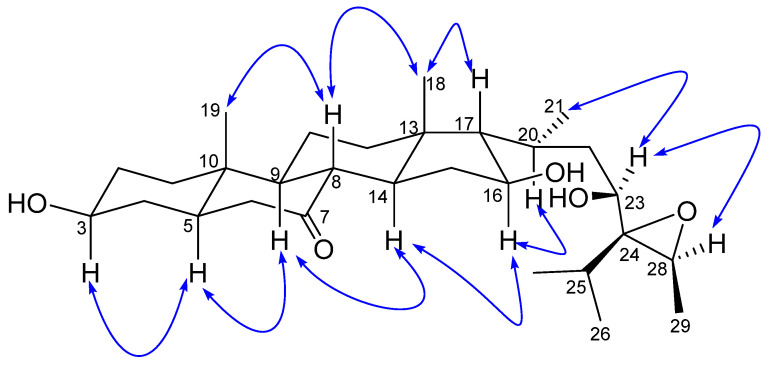
Key ROESY correlations of kotschyanoside A (**1**).

**Figure 4:**
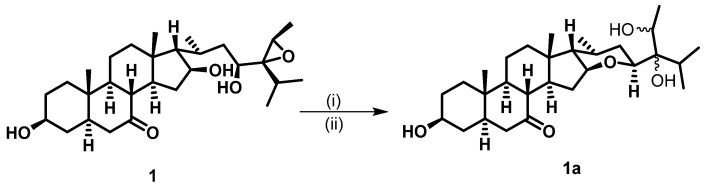
Cyclization of **1** to **1a** under conditions if (i) *p*-TsOH (50 mg) and (ii) DMF overnight at 90 °C.

The relative stereochemistry of **1** was established by exploring its ROESY spectrum. Stigmastanes are known to occur in a chair–chair–chair–boat conformer. As a result, the hydroxyl at C-3 was found to be oriented equatorially because of the cross-peak between H-3 and the characteristic H-5 positioned axially. The proton H-17 was suggested to be *trans*-positioned to H-16 due to the absence of a cross-peak between the two protons in the ROESY spectrum. Further ROESY interactions evidenced correlations between H-21 (biosynthetically *α*-oriented) and H-23 and H-28 (Figure 3). The isopropyl moiety was *β*-oriented as in all stigmastanes (Supplementary Materials, Figures S1–S9). The position and stereochemistry of the hydroxyls at C-16 and C-23 were further verified through the cyclization reaction of **1** under an acidic condition (Figure 4). Only pyran or furan are expected when both hydroxyls are in the same side of the plane. Accordingly, the C-16-O-C-23 oxanes (**1a**) with an opened epoxide at C-24/C-28 was isolated from the reaction mixture. Its NMR data (Figures S11–S16) as well as its mass spectrum (Figure S10) were completely consistent with the expected structure. In fact, its HMBC spectrum showed a strong cross-peak between H-16 (δ_H_ 3.95) and C-23 (δ_C_ 75.7) establishing the oxane ring moiety. In its ^13^C NMR spectrum, the characteristic signals at δ_C_ 69.3 and 57.2 for the epoxy group disappeared, and the signals for C-24 and C-28 were observed at δ_C_ 76.5 and 70.6, respectively. Thus, the structure of compound **1a** no longer contained an epoxy group. As a consequence, compound **1** was thus fully characterized as 24,28-epoxy-3,16,23-trihydroxy stigmastan-7-one, for which we proposed the trivial name kotschyanoside A. 

Compound **2** was isolated as a white amorphous powder with an ${\left[\alpha \right]}_{D}^{20}$ of −11.5 (*c* 0.5, MeOH). Its molecular formula (C_29_H_48_O_6_) was determined via HR-ESI-MS, which showed a sodium adduct ion peak [M+Na]^+^ at *m/z* 515.3349 (calcd. for C_29_H_48_O_6_Na^+^, 515.3343). The molecular mass was 16 Da higher compared to **1**, suggesting an additional hydroxyl group present in **2.** Its IR spectrum showed absorption bands corresponding to hydroxyl (3392 cm^−1^), carbonyl (1707 cm^−1^), and aliphatic chains (2939 and 2871 cm^−1^). Its NMR data were superimposable on those of compound **1** except for the occurrence of a hydroxylated methine at *δ_H_*/*δ_C_* 3.85/74.6 (H-6) in **2** instead of a methylene (*δ_H_* 2.10/*δ_C_* 46.8) in **1** (Table 1 and Table 2). The HMBC spectrum of **2** evidenced an important cross-peak from H-6 to a ketone group at *δ_C_* 211.7. Further interactions from H-6 to C-4 (*δ_C_* 33.4), C-5 (*δ_C_* 53.7), and C-10 (*δ_C_* 36.1) confirmed the position of the new hydroxyl group at C-6. The relative stereochemistry of **2** was also established by means of its ROESY spectrum as identical to that of compound **1**. The ROESY cross-peak observed from H-19 (positioned equatorially) to H-6 implied that the hydroxyl at C-6 was positioned axially (Supplementary Materials, Figures S17–S24). Thus, **2** was identified as 24,28-epoxy-3,6,16,23-tetra-hydroxystigmastan-7-one, for which we proposed the trivial name kotschyanoside B.

Compound **3** was obtained as a white amorphous powder with an ${\left[\alpha \right]}_{D}^{20}$ of −36.8 (*c* 1.0, MeOH). Its molecular formula (C_35_H_58_O_10_) was determined via HR-ESI-MS, which showed a sodium adduct ion peak [M+Na]^+^ at *m/z* 661.3928 (calcd. for C_35_H_58_O_10_Na^+^, 661.3922). The molecular mass was 162 Da higher compared to **1**, suggesting the presence of an additional hexose moiety in **3**. Its IR spectrum showed characteristic absorption bands corresponding to hydroxyl (3356 cm^−1^), carbonyl (1705 cm^−1^), and aliphatic chains (2935 and 2871 cm^−1^). Its NMR data were similar to those of **1** except for the presence of a glucosyl group (*δ_C_* 102.3, 78.1, 77.9, 75.2, 71.0, and 62.8), which accounted for the additional one degree of unsaturation and was identified as β-D-glucopyranoside via comparison of its NMR data with reported values [8,25]. The position of the sugar was defined based on the HMBC interaction from the anomeric proton H-1’ (*δ_H_* 4.40) to *δ_C_* 69.3 (C-3), which was further confirmed by the glycosidation shifts in the resonances of C-2, C-3, and C-4 (*δ_C_* 35.3, 78.6, and 47.0, respectively) as compared to their respective values for compounds **1** and **2** (Table 1). The relative stereochemistry of **3** was consistent with those of **1** and **2** when comparing their ROESY data (Supplementary Materials, Figures S25–S33). Thus, **3** was fully characterized as 24,28-epoxy-3,16,23-trihydroxystigmastan-7-one 3-*O*-*β*-D-glucopyranoside, for which we proposed the trivial name kotschyanoside C. 

Compound **4** was isolated as a white amorphous powder with an ${\left[\alpha \right]}_{D}^{20}$ of −26.7 (*c* 1.0, MeOH). Its molecular formula (C_35_H_58_O_11_) was determined via HR-ESI-MS, which showed a sodium adduct ion peak [M+Na]^+^ at *m/z* 677.3879 (calcd. for C_35_H_58_O_11_Na^+^, 677.3871). The molecular mass was 162 Da higher compared to **2**, suggesting the presence of an additional hexose moiety in **4**. Its IR spectrum showed characteristic absorption bands corresponding to hydroxyl (3354 cm^−1^), carbonyl (1704 cm^−1^), and aliphatic chains (2935 and 2871 cm^−1^). The NMR data of **4** were closely related to those of **3** except for the signal of an additional oxygenated methine at *δ_C_*/*δ_H_* 76.5/3.99, which was located at C-6 due to HMBC interactions from the latter to carbons C-8 (48.4), C-5 (*δ_C_* 55.2), and C-7 (*δ_C_* 212.3). The relative stereochemistry of **4** was consistent with that of **3** when comparing the ROESY data of both **3** and **4** (Supplementary Materials, Figures S34–S41). Thus, **4** was fully characterized as 24,28-epoxy-3,6,16,23-tetrahydroxystigmastan-7-one 3-*O*-*β*-D glucopyranoside, for which we proposed the trivial name kotschyanoside D. 

The other isolates were identified via comparison of their spectroscopic data with those reported in the literature as quercetin (**5**) [27], apigenin (**6**) [10], and a mixture of *β*-sitosterol 3-*O*-*β*-D-glucopyranoside (**7**) and stigmasterol 3-*O*-*β*-D-glucopyranoside (**8**) [28].

### 2.2. Chemophenetic Significance

Overall, eight (**1**–**8**) compounds were isolated during the chemical investigations of this species, including new stigmastane-type glycosides with unique oxygenated patterns (**1**–**4**), two flavonoids (**5**–**6**), and a mixture of two readily available phytosterols (**7**–**8**). Sesquiterpenoid and stigmastane-type steroids are the chemophenetic markers of the genus *Vernonia* [10,29,30]. To the best of our knowledge, this is the third report disclosing the chemical constituents of the studied species. Our investigation allowed us to postulate that sesquiterpenoid lactones were not occurring in *V. kotschyana* after phytochemical screening tests and LC-MS facilities were used.

The new compounds displayed a new oxygenated pattern in stigmastane-type steroids found in *Vernonia*. Stigmastanes have been highlighted in some species of the genus, including *V. guineesis* and *V. amygdalina*. Mainly, the side chain of *Vernonia* steroids usually bears a γ-lactone fused to a tetrahydrofuran group resulting from successive oxidation and cyclization processes [4,17]. The new compounds **1***–***4** could have resulted from the hydrolysis of this well-known moiety in *Vernonia* species. Moreover, our findings revealed new oxygenated carbons in the steroid skeleton, namely positions C-6 and C-7, indicating that steroids from *V. kotschyana* originate instead from the stigmasterol backbone. This might represent a new significant chemophenetic marker for plants of the genus *Vernonia*.

Although flavonoids have been reported in Vernonia species (including *V. cinerascens* [11] and *V. cinarea* [31] for compound **5** and *V. amygdalina* for compound **6** [32]), they are herein reported for the first time from V. kotschyana. This observation provides new insights into the occurrence of flavonoids in the genus *Vernonia* and the family Asteraceae. 

### 2.3. Biological Results

The antibacterial activities of the crude extract and major fractions (FA–FD) obtained from the main column chromatography, as well as pure compounds obtained in sufficient amounts (**1**–**3**), were evaluated against five highly prevalent bacterial strains: *Escherichia coli* ATCC25322, *Staphylococcus aureus* ATCC25923, *Staphylococcus pneumoniae* ATCC461916, *Pseudomonas aeruginosa* HM801, and the clinical strain *Klebsiella pneumonia*. The extract and three fractions (FA, FC, and FD) showed weak activity (with MIC > 500 µg/mL), whereas fraction FB was moderately active on *S. aureus* ATCC25923 (MIC = 250 µg/mL) and *P. aeruginosa* HM801 (MIC = 250 µg/mL) and moderate on the clinical strain *K. pneumonia* (MIC = 500 µg/mL). Compounds **1** and **2** displayed moderate activity (125 < MIC < 500 µg/mL) against the tested strains, while compound **3** was totally inactive at the tested concentration.

The biological properties of stigmastane-type steroids and flavonoids are well documented [18], and the presence of both classes of compounds in an extract might strengthen evidence of the medicinal value of V. *kotschyana* (mainly as potential inhibitors of *Helicobacter pylori*) for a possible mode of action of the plant in the treatment of gastric ulcer.

## 3. Experimental

### 3.1. General Experimental Procedures

Electrospray ionization (ESI) data were obtained on a 1200-series HPLC system or a 1260-series Infinity II HPLC system (Agilent-Technologies, Santa Clara, CA, USA) with a binary pump and integrated diode array detector coupled to an LC/MSD-Trap-XTC-mass spectrometer (Agilent-Technologies) or an LC/MSD Infinity lab LC/MSD (G6125B LC/MSD). High-resolution mass spectra were recorded on a Micromass-Q-ToF-Ultima-3 mass spectrometer (Waters, Milford MA, USA) with a LockSpray interface and a suitable external calibrant using gradients of acetonitrile–water (containing 0.1% formic acid) as the elution system. Infrared (IR) spectra were recorded on an FTIR spectrometer (Bruker Tensor 27) equipped with a diamond ATR unit; the frequency of the absorption is reported in cm^−1^. NMR data were obtained on a Bruker Avance III 500 HD (^1^H: 500 MHz, ^13^C: 125 MHz) or Avance 600 (^1^H: 600 MHz, ^13^C: 150 MHz); chemical shifts *δ* (ppm) were referenced relative to the residual solvent signal and/or tetramethylsilane (TMS).

Compounds were purified via chromatographic methods using silica gel (35–70 μm, Acros Organics, Waltham, MA, USA) and Sephadex LH-20 automated column chromatography on a Büchi Reveleris^®^ X2 with a binary pump and an ELSD detector using flash columns or a Biotage Snap Ultra C18 column with a gradient at various flow rates. Thin-layer chromatography (TLC) was carried out on silica plates (TLC Silica 60 F_254_ by Merck, Darmstadt, Germany), and spots were detected by spraying with 20% H_2_SO_4_ followed by charring at 100 °C. 

### 3.2. Plant Material

The whole plant of *V. kotschyana* was collected in November 2016 at Bamendjing (Mbouda Subdivision), Western Region of Cameroon, and identified by Victor Nana, a retired botanist of the National Herbarium of Cameroon, where a voucher specimen (N° 48782 HNC) was deposited.

### 3.3. Extraction and Isolation 

The whole plant of *V. kotschyana* (3.7 kg) was macerated in a mixture of CH_2_Cl_2_/MeOH (1:1) at room temperature for 48 h, and the extraction process was conducted thrice. The resulting solution was filtered, and the removal of the solvent in vacuo afforded 100.1 g of a semi-solid crude extract. Part of the crude extract (90.5 g) was subjected to silica gel (230–400 mesh) column chromatography using a stepwise gradient of EtOAc in petroleum ether (PE) and MeOH in EtOAc. A total of 95 fractions of 250 mL each were collected and combined based on TLC profiles into 4 main fractions (FA–FD). Fraction FA (2.5 g, PE) was mainly fatty content and was not further investigated. Fractions FB (10 g, PE/EtOAc, 95:5–9:1 *v*/*v*) and FC (17.2 g, PE/EtOAc, 9:1–8.5: 1.5 *v*/*v*) were combined based on their TLC profiles and labeled FBC (27.2 g). A mass of 27.1 g of this fraction was submitted to open column chromatography over silica gel (70–230 mesh) and eluted with a gradient of acetone in PE to yield compound **1** (1.2 g). The resulting fractions were grouped into three subfractions (FBC1–FBC3) based on their TLC profiles. Subfraction FBC2 (3 g, PE/acetone, 8.5:1.5–7:3 *v*/*v*) was further chromatographed over silica gel and eluted with a gradient of acetone in PE to yield compounds **5** (3.0 mg) and **2** (4.8 mg). Fractions FD (55 g, PE/acetone, 95:5–9:1 *v*/*v*) was submitted to open column chromatography over silica gel (70–230 mesh) and eluted with a gradient of acetone in PE to yield compound **3** (50.7 mg). The resulting fractions were grouped into four subfractions (FD-S1–FD-S4) based on their TLC profiles. Subfraction FD-S1 (15.6 mg, PE/acetone 3:2–1:1 *v*/*v*) was chromatographed over silica gel and eluted with a gradient of acetone in PE to yield compound **6** (2.5 mg). Subfraction FD-S2 (6.3 g, PE/acetone 3:2–1:1 *v*/*v*) was chromatographed over silica gel and eluted with a gradient of acetone in PE to obtain the mixture of compounds **7** and **8**. Subfractions FD-S3 and FD-S4 (40.2 g, PE/EtOAc 3:2–1:1 *v*/*v*) were combined based on their TLC profile and labeled FD-S3, which was purified over a silica gel column and eluted with a gradient of acetone in PE to afford compounds **3** (1.2 g) and **4** (5.3 mg). 

### 3.4. Kotschyanoside A *(**1**)*

White amorphous powder; ${\left[\alpha \right]}_{D}^{20}$ −23, (*c* 0.5, MeOH); IR (ATR) *υ*_max_ 3392, 2940, 2870, 1760 cm^−1^; ^1^H and ^13^C NMR data, see Table 1 and Table 2; (+)-HRESIMS *m/z* 499.3386 [M+Na]^+^ (calcd. for C_29_H_48_O_5_Na^+^, 499.3399).

### 3.5. Kotschyanoside B *(**2**)*

White amorphous powder; ${\left[\alpha \right]}_{D}^{20}$ −11.5 (*c* 0.5, MeOH); IR (ATR) *υ*_max_ 3392, 2939, 2871, 1707 cm^−1^; ^1^H and ^13^C NMR data, see Table 1 and Table 2; (+)-HRESIMS *m/z* 515.3349 [M+Na]^+^ (calcd. for C_29_H_48_O_6_Na^+^, 515.3349).

### 3.6. Kotschyanoside C *(**3**)*

White amorphous powder ${\left[\alpha \right]}_{D}^{20}$ −36.8 (*c* 1.0, MeOH); IR (ATR) *υ*_max_ 3356, 2935, 2871, 1705 cm^−1^; ^1^H and ^13^C NMR data, see Table 1 and Table 2; (+)-HRESIMS *m/z* 661.3928 [M+Na]^+^ (calcd. for C_35_H_58_O_10_Na^+^, 661.3922).

### 3.7. Kotschyanoside D *(**4**)*

White amorphous powder ${\left[\alpha \right]}_{D}^{20}$ −26.7 (*c* 1.0, MeOH); IR (ATR) *υ*_max_ 3354, 2935, 2871, 1704 cm^−1^; ^1^H and ^13^C NMR data, see Table 1 and Table 2; (+)-HRESIMS *m/z* 677.3879 [M+Na]^+^ (calcd. for C_35_H_58_O_11_Na^+^, 677.3871).

### 3.8. Cyclization of Compound ***1***

A total of 50.0 mg of compound **1** was mixed with *p*-TsOH (50.0 mg), dissolved in 2 mL of DMF, and stirred for 24 h (overnight) at 90 °C. The solvent was evaporated, and the residual was purified via column chromatography followed by MPLC with a gradient of EtOAc in PE to afford compound **1a** (5.6 mg) along with an unresolved complex mixture. 

Compound **1a**: white amorphous powder ^1^H NMR (600 MHz, acetone-*d*_6_) *δ*_H_ 3.97–3.93 (m, 1H), 3.74 (dd, *J* = 7.8, 4.8 Hz, 1H), 3.62 (dq, *J* = 8.4, 6.7 Hz, 1H), 3.43 (d, *J* = 8.4 Hz, 1H), 2.41 (t, *J* = 11.3 Hz, 1H), 2.35–2.27 (m, 1H), 1.87 (ddd, *J* = 10.4, 7.5, 5.1 Hz, 1H), 1.83–1.75 (m, 2H), 1.65 (dddd, *J* = 28.8, 13.3, 5.6, 3.2 Hz, 3H), 1.46 (dddd, *J* = 26.2, 12.2, 5.1, 2.6 Hz, 2H), 1.10 (d, *J* = 6.7 Hz, 3H), 1.01 (s, 3H), 1.00–0.97 (m, 1H), 0.95 (d, *J* = 6.8 Hz, 3H), 0.93–0.86 (m, 2H), 0.84 (d, *J* = 6.9 Hz, 3H), 0.77 (d, *J* = 7.2 Hz, 3H), 0.76 (d, *J* = 4.7 Hz, 0H), 0.74 (s, 3H). ^13^C NMR (150 MHz, Acetone-*d*_6_) *δ*_C_ 77.1, 76.5, 75.7, 70.6, 69.8, 59.7, 55.7, 55.5, 48.8, 46.6, 45.9, 45.6, 42.1, 38.1, 37.8, 36.1, 36.0, 34.6, 31.8, 31.1, 30.0, 24.3, 22.0, 21.2, 19.9, 18.7, 17.5, 17.2, 14.4, 13.6, 11.1. (+)-ESIMS *m/z* 499.36 [M+Na]^+^.

### 3.9. Bioassay

Five pathogenic bacterial strains were used. The antibacterial assay was performed on five bacteria strains including four standards (*Escherichia coli* ATCC25322, *Staphylococcus aureus* ATCC25923, *Staphylococcus pneumoniae* ATCC461916, and *Pseudomonas aeruginosa* HM801) and the clinical *Klebsiella pneumonia* CPC. They were maintained on fresh Mueller Hinton Agar (MHA) for 24 h prior to any antibacterial assay, and the minimal inhibitory concentrations (MICs) of the tested samples were determined as reported previously [33].

## 4. Conclusions

The chemical investigation of *Vernonia kotschyana* led to the isolation of four new stigmastane-type glycosides with unique oxygenated patterns (**1**–**4**). Compound **1**, one of the most active metabolites against the tested microbial strains (125–250 µg/mL), was isolated from the most active fraction, FB (250 μg/mL). These results indicated that *V. kotschyana* shares similar chemical characteristics with other species of this genus and might provide additional insights into the chemotaxonomic classification of the genus *Vernonia*. The discovery of more steroids featuring similar chemical diversities as encountered for compounds **1**–**4** from previously unstudied *Vernonia* species might provide more information on the suggested occurrence pattern highlighted in the present report. Such cues would trigger the development of strategies to clear up the biosynthesis of steroids as they occur in the *Vernonia* genus. Ultimately, the recorded bioactivity is an indication to look further toward the validation of health benefits associated with the plant. 

## Figures and Tables

**Table 1:** ^13^C NMR spectroscopic data for compounds **1**–**4** (*δ* in ppm).

Position	1 ^a^	2 ^b^	3 ^c^	4 ^c^
	***δ*c**	***δ*c**	***δ*c**	***δ*c**
1	36.7	36.1	37.1	37.6
2	32.1	30.8	35.3	37.5
3	70.7	69.3	78.6	78.7
4	39.1	33.4	47.0	30.6
5	47.5	53.7	46.7	55.2
6	46.8	74.6	30.1	76.5
7	212.2	211.7	214.4	212.3
8	50.1	46.8	50.6	48.4
9	55.9	54.6	48.2	56.7
10	36.9	35.8	37.3	49.6
11	22.1	21.0	22.5	22.6
12	39.1	38.3	40.1	40.0
13	43.1	41.7	43.5	43.4
14	47.8	46.3	48.2	48.0
15	37.4	35.9	37.2	36.8
16	72.3	70.6	73.0	73.0
17	62.0	60.2	62.1	62.0
18	13.8	12.9	13.5	13.4
19	12.2	12.7	12.0	13.3
20	28.1	26.5	28.3	28.3
21	20.7	19.5	20.4	20.3
22	40.9	38.3	40.6	40.5
23	69.7	67.5	69.4	69.5
24	69.3	67.7	70.0	70.0
25	30.8	29.4	31.2	31.1
26	19.0	18.1	18.5	18.5
27	19.6	18.8	19.1	19.1
28	57.2	55.5	58.0	58.0
29	14.2	13.3	13.7	13.7
1’	-	-	102.3	102.3
2’	-	-	75.2	75.1
3’	-	-	78.1	78.1
4’	-	-	77.9	77.9
5’	-	-	71.0	71.6
6’	-	-	62.8	62.7

^a^ Pyridine-*d*_5_; ^b^ DMSO-*d*_6_; ^c^ MeOH-*d*_4_.

**Table 2:** ^1^H NMR spectroscopic data for compounds **1**–**4** (*δ* in ppm).

Position	1 ^a^	2 ^b^	3 ^c^	4 ^c^
	*δ*_H_	*δ*_H_	*δ*_H_	*δ*_H_
1	1.70, m	0.92, m	0.87, m	1.07, m
2	2.12, m 1.76, m	1.63, m	1.76, m	1.80, m
3	3.85, m	3.27, m	3.76, m	3.76, m
4	1.83, m 1.69, m	1.99, m	2.51 (t, 12.8)	2.35, m
5	1.47, m	1.07, m	2.58, m	124, m
6	2.10, m	3.85, m	1.95, m	3.99 (d, 11.9)
7	-	-	-	-
8	2.41 (t, 11.0)	2.52, br s	2.58 (t, 11.3)	2.63 (t, 11.4)
9	1.08, m	0.95, m	1.49, m	1.11, m
10	-	-	-	-
11	1.50, m	1.46, m	1.59, m	1.61, m
12	2.00, m	1.86, m	2.02, m 1.11, m	2.03, m 1.11, m
13	-	-	-	-
14	1.47, m	1.18, m	1.49, m	1.38, m
15	3.26, m	2.80, m	2.80, m 1.06, m	2.81, m 1.09, m
16	4.67 (td, 7.6, 4.3)	4.23, m	4.39, m	4.39, m
17	0.96, m	0.90, m	1.04, m	2.82, m
18	1.06, s	0.80, s	0.91, s	0.92, s
19	1.09, s	1.04, s	1.15, s	1.20, s
20	2.47, m	1.89, m	2.02, m	2.03, m
21	1.03 (d, 6.6)	0.95 (d, 6.6)	1.08 (d, 6.4)	1.08 (d, 7.0)
22	1.45, m	-	1.58, m 1.12, m	1.57, m 1.10, m
23	4.28, br d	3.73, m	3.92 (dd, 11.2, 1.6)	3.92 (dd, 11.2, 1.6)
24	-	-	-	-
25	1.82, m	1.64, m	1.75, m	1.74, m
26	1.10 (d, 7.0)	1.00 (d, 1.7)	1.11 (d, 2.5)	1.10 (d, 2.3)
27	1.14 (d, 7.2)	0.99 (d, 1.5)	1.09 (d, 2.1)	1.09 (d, 1.9)
28	3.51 (q, 5.5)	3.08 (q, 5.6)	3.24 (q, 5.7)	3.25 (q, 5.7)
29	1.37 (d, 5.6)	1.22 (d, 5.6)	1.33 (d, 5.7)	1.33 (d, 5.7)
1’	-	-	4.40 (d, 7.8)	4.40 (d, 7.8)
2’	-	-	3.18, m	3.35, m
3’	-	-	3.36, m	3.36, m
4’	-	-	3.27, m	3.27, m
5’	-	-	3.29, m	3.28, m
6’	-	-	3.85, m 3.65 (d, 5.1)	3.87 (dd, 11.9, 1.6) 3.65, m

^a^ Pyridine-*d*_5_; ^b^ DMSO-*d*_6_; ^c^ MeOH-*d*_4._ s = singlet; d = doublet; t = triplet; q = quartet; m = multiplet; dd = doublet of doublet; td = triplet of doublet; br d = broad doublet; br s = broad singlet.

## Data Availability

Not applicable.

## Supplementary Materials

The following supporting information can be downloaded at: https://www.mdpi.com/article/10.3390/molecules28135278/s1: NMR, UV, IR, and HR-ESIMS data for compounds **1**−**4** (Figures S1–S9 and S17–S41); NMR and ESIMS spectra of the derivative **1a** (Figures S10–S16); 1D NMR spectra of known compounds **5**–**8** (Figures S42–S45).

## Sample Availability

Samples of the compounds are available from the authors.

## References

[1] Akinpelu A.D. (1999). Antimicrobial activity of *Vernonia amygdalina* leaves. Fitoterapia.

[2] Koshimizu K., Ohigashi H., Huffman M.A. (1994). Use of *Vernonia amygdalina* by wild chimpanzee: Possible roles of its bitter and related constituents. Physiol. Behav..

[3] Donfack N.R.A., Toyang J.N., Wabo K.H., Tane P., Awouafack D.M., Kikuchi H., Tamokou J.D.D., Kuiate J.R., Oshima Y. (2012). Stigmastane derivatives from the roots of *Vernonia guineensis* and their antimicrobial activity. Phytochem. Lett..

[4] Ohigashi H., Jisaka M., Takagaki T., Nozaki H., Tada T., Huffman M.A., Nishida T., Kaji M., Koshimizu K. (1991). Bitter principle and a related steroid glucoside from *V. amygdalina*, a possible medicinal plant for wild chimpanzees. Chem. Biol. Technol. Agric..

[5] Jisaka M., Kawanaka M., Sugiyama H., Takegawa K., Huffman M.A., Ohigashi H., Koshimizu K. (1992). Antischistosomal activities of sesquiterpene lactones and steroid glucosides from *Vernonia amygdalina*, possibly used by wild chimpanzees against parasite-related diseases. Biosci. Biotechnol. Biochem..

[6] Jisaka M., Ohigashi H., Takagaki T., Nozaki H., Tada T., Hirota M., Irie R., Huffman M.A., Nishida T., Kaji M. (1992). Bitter steroid glucosides, vernosides A1, A2, A3 and related B1 from a possible medicinal plant *Vernonia amygdalina* used by wild chimpanzees. Tetrahedron.

[7] Tchinda A.T., Tsopmo A., Tane P., Ayafor F.J., Connolly J.D., Sterner O. (2002). Vernoguinosterol and vernoguinoside, trypanocidal stigmastane derivatives from *Vernonia guineensis* (Asteraceae). Phytochemistry.

[8] Anh H.L.T., Vinh L.B., Lien L.T., Cuong V.P., Arai M., Ha P.T., Lin N.H., Dat H.T.T., Cuong V.L.C., Kim H.Y. (2021). In vitro study on a-amylase and a-glucosidase inhibitory activities of a new stigmastane-type steroid saponin from the leaves of *Vernonia amygdalina*. Nat. Prod. Res..

[9] Bitchagno M.T.G., Nchiozem-Ngnitedem V.-A., Wandji T.N., Noulala T.G.C., Fobofou T.A.S., Lenta N.B., Sharma K., Mishra K., Kamal Senapati K., Danciu C. (2021). Plant-Derived Compounds against Microbial Infections and Cancers. Bioactive Compounds in Nutraceutical and Functional Food for Good Human Health.

[10] Bitchagno G.T.M., Koffi G.J., Simo K.I., Kagho D.U.K., Ngouela A.S., Lenta N.B., Sewald N. (2021). LC-ToF-ESI-MS patterns of hirsutinolide-like sesquiterpenoids present in the *Elephantopus mollis* Kunth extract and chemophenetic significance of its chemical constituents. Molecules.

[11] Toyang J.N., Verpoorte R. (2013). A review of the medicinal potentials of plants of the genus *Vernonia* (Asteraceae). J. Ethnopharmacol..

[12] Nergard C.S., Matsumoto T., Inngjerdingen M., Inngjerdingen K., Hokputsa S., Harding S.E., Michaelsen T.E., Diallo D., Kiyohara H., Paulsen B.S. (2005). Structural and immunological studies of a pectin and a pectic arabinogalactan from Vernonia kotschyana Sch. Bip. ex Walp. (Asteraceae). Carbohydr. Res..

[13] Diarra M.L., Denou A., Coulibaly L.B., Togola A., Sanogo D., Sanogo R., Traore M., Diallo D., Noba K. (2018). Caractéristiques botaniques et phytochimiques de *Vernonia kotschyana* Sch. Bip. ex Walp. mise en culture et utilisée dans le traitement des gastrites et l’ulcère gastroduodénal au Mali. Int. J. Biol. Chem. Sci..

[14] Nergard C.S., Diallo D., Michaelsen T.E., Malterud K.E., Kiyohara H., Matsumoto T., Yamada H., Paulsen B.S. (2004). Isolation, partial characterization and immunomodulating activities of polysaccharides from *Vernonia kotschyana* Sch. Bip. ex Walp. J. Ethnopharmacol..

[15] Nergard C.S., Kiyohara H., Reynolds J.C., Thomas-Oates J.E., Matsumoto T., Yamada H., Michaelsen T.E., Diallo D., Paulsen B.S. (2005). Structure-immunomodulating activity relationships of a pectic arabinogalactan from *Vernonia kotschyana* Sch Bip. ex Walp. Carbohydr. Res..

[16] Focho D.A., Ndam W.T., Fonge B.A. (2008). Medicinal plants of Aguambu—Bamumbu in the Lebialem highlands, SouthWest province of Cameroon. Afr. J. Pharm. Pharmacol..

[17] Vasincua A., Luca V.S., Charalambous C., Neophytou M.C., Skalicka-Woźniakg K., Mironc A. (2022). LC-HRMS/MS phytochemical profiling of *Vernonia kotschyana* Sch. Bip. ex Walp.: Potential involvement of highly-oxygenated stigmastane-type saponins in cancer cell viability, apoptosis and intracellular ROS production. S. Afr. J. Bot..

[18] Sanogo R., Germano M.P., Tommasi D.N., Pizza C., Aquino R. (1997). Vernoniosides and an androstane glycoside from *Vernonia kotschyana*. Phytochemistry.

[19] Inngjerdingen T.K., Thöle C., Diallo D., Paulsen S.B. (2014). Andreas Hensel, A. Inhibition of *Helicobacter pylori* adhesion to human gastric adenocarcinoma epithelial cells by aqueous extracts and pectic polysaccharides from the roots of *Cochlospermum tinctorium* A. Rich. and *Vernonia kotschyana* Sch. Bip. ex Walp. Fitoterapia.

[20] Tameye N.S.J., Akak M.C., Tabekoueng B.G., Mkounga P., Bitchagno M.T.G., Lenta N.B., Sewald N., Nkengfack E.A. (2021). Chemical constituents from *Diospyros fragrans* Gürke (Ebenaceae). Biochem. Syst. Ecol..

[21] Wandji T.N., Bitchagno G.T.M., Tchamgoue J., Stammler H.-G., Frese M., Lenta B.N., Sewald N., Kouam F.S. (2022). Furanocoumarins and other constituents from the twigs of *Ficus chlamydocarpa* Mildbraed & Burret (Moraceae). Phytochem. Lett..

[22] Wouamba N.S.C., Happi M.G., Lenta N.B., Sewald N., Kouam F.S. (2020). Vernoguinamide: A new ceramide and other compounds from the root of *Vernonia guineensis* Benth. and their chemophenetic significance. Biochem. Syst. Ecol..

[23] Ambadiang M.M.M., Atontsa K.C.B., Tankeo B.S., Nayim P., Wamba N.E.B., Bitchagno M.T.G., Mpetga S.D.J., Penlap B.V., Kuete V. (2020). Bark extract of *Cassia sieberiana* DC. (Caesalpiniaceae) displayed good antibacterial activity against MDR gram-negative phenotypes in the presence of phenylalanine-arginine β-naphthylamide. BMC Complement. Med. Ther..

[24] Hua L., Qi W.Y., Hussain S.H., Gao K., Arfan M. (2012). Highly oxygenated stigmastane-type steroids from the aerial parts of *Vernonia anthelmintica* Willd. Steroids.

[25] Igile G., Oleszek W., Jurzysta M. (1995). Vernoniosides D and E, two novel saponins from *Vernonia amygdalzna*. J. Nat. Prod..

[26] Cioffi G., Sanogo R., Diallo D.R.G., Tommasi D.N. (2004). New Compounds from an extract of *Vernonia colorata* leaves with anti-inflammatory Activity. J. Nat. Prod..

[27] Huang W., Wan C., Zhou S. (2013). Quercetin—A flavonoid compound from *Sarcopyramis bodinieri* var delicate with potential apoptotic activity in HepG2 Liver Cancer Cells. Trop. J. Pharm. Res..

[28] Rai N.P., Adhikari B.B., Paudel A., Masuda K., Mckelvey R.D., Manandhar M.D. (2006). Phytochemical constituents of the flowers of *Sarcococca coriacea* of Nepalese origin. J. Nepal Chem. Soc..

[29] King B., Jone B.S. (1982). Chemosystematics of *Vernonia* series Flexuosae (Vernonieae: Compositae). Bull. Torrey Bot. Club.

[30] Mabry T.J., Abdel-Base Z., Padolina G., Jones S.B. (1975). Systematic implications of flavonoids and sesquiterpene lactones in species of *Vernonia*. Biochem. Syst. Ecol..

[31] Youn J.U., Miklossy G., Chai X., Wongwiwatthananukit S., Toyama O., Thanapat S., Turkson J., Chang L.C. (2014). Bioactive sesquiterpene lactones and other compounds isolated from *Vernonia cinerea*. Fitoterapia.

[32] Dagnon S., Novkova Z., Bojilov D., Nedialkov P., Kouassi C.H. (2019). Development of surrogate standards approach for the determination of polyphenols in *Vernonia amygdalina* Del. J. Food Compost. Anal..

[33] Nguengang R.T., Tchegnitegni B.T., Nono E.C.N., Bellier Tabekoueng G., Fongang Y.S.F., Bankeu J.J.K., Chouna J.R., Nkenfou C.N., Fekam F.B., Sewald N. (2023). Constituents of the Stem Bark of *Symphonia globulifera* Linn. f. with Antileishmanial and Antibacterial Activities. Molecules.

