# Supplementary material for: Polyoxygenated Stigmastane-Type Steroids from Vernonia kotschyana Sch. Bip. ex Walp. and Their Chemophenetic Significance
Source: Molecules. 2023 Jul 7;28(13):5278. doi: 10.3390/molecules28135278 (PMC10343579; doi:10.3390/molecules28135278)
Supplement: Supplementary file 1 [file molecules-28-05278-s001.zip › molecules-2441761-supplementary.pdf]

Polyoxygenated stigmastane-type steroids from *Vernonia kotschyana* Sch. Bip. ex Walp. and their chemophenetic significance†

Nadine Tseme Wandji[1,2,3], Gabin Thierry M. Bitchagno[3,4], Isabelle Mawabo Kamga[5], Joseph Tchamgoue[1,2], Celine Nguefeu Nkenfou[5,6], Bruno Ndjakou Lenta[1], Norbert Sewald[3,*], Simeon Fogue Kouam[1,*]

[1]Department of Chemistry, Higher Teacher Training College, University of Yaounde I, P.O. Box 47, Yaounde, Cameroon.

[2]Department of Organic Chemistry, Faculty of Science, University of Yaounde I, P.O. Box 812, Yaounde, Cameroon

[3]Organic and Bioorganic Chemistry, Faculty of Chemistry, Bielefeld University, D-33501 Bielefeld, Germany

[4]Department of Chemistry, University of Dschang, P.O. Box 67 Dschang, Cameroon

[5]Department of Biology, Higher Teacher Training College, University of Yaounde I, P.O. Box 47, Yaounde, Cameroon

[6]Molecular Biology Center, P. O. Box 14495 Yaounde, Cameroon

Content

Figure S1: IR spectrum of compound **1** 3
Figure S2: ESI-HR mass spectrum of compound **1** 3
Figure S3: ^{1}H NMR ($CDCl_3$, 500 MHz) spectrum of compound **1** 4
Figure S4: ^{13}C NMR ($CDCl_3$, 125 MHz) spectrum of compound **1** 4
Figure S5: DEPT 135 spectrum of compound **1** 5
Figure S6: HSQC spectrum of compound **1** 5
Figure S7: HMBC spectrum of compound **1** 6
Figure S8: COSY spectrum of compound **1** 6
Figure S9: ROESY spectrum of compound **1** 7
Figure S10: ESI-HR mass spectrum of compound **1a** 7
Figure S11: ^{1}H NMR (acetone-d_6, 600 MHz) spectrum of compound **1a** 8
Figure S12: ^{13}C NMR (acetone-d_6, 150 MHz) spectrum of compound **1a** 8
Figure S13: COSY spectrum of compound **1a** 9
Figure S14: HSQC spectrum of compound **1a** 9

Figure S15: HMBC spectrum of compound **1a** 10
Figure S16: NOESY spectrum of **1a** 10
Figure S17: ESI-HR mass spectrum of compound **2** 11
Figure S18: IR spectrum of compound **2**........ 11
Figure S19: ^{1}H NMR ($CDCl_3$, 500 MHz) spectrum of compound **2** 12
Figure S20: ^{13}C NMR ($CDCl_3$, 125 MHz) spectrum of compound **2**........ 12
Figure S21: DEPT 135 spectrum of compound **2** 13
Figure S22: HMBC spectrum of compound **2** 13
Figure S23: COSY spectrum of compound **2**........ 14
Figure S24: ROESY spectrum of compound **2** 14
Figure S25: ESI-HR mass spectrum of compound **3** 15
Figure S26: IR spectrum of compound **3**........ 15
Figure S27: ^{1}H NMR ($CDCl_3$, 600 MHz) spectrum of compound **3** 16
Figure S28: ^{13}C ($CDCl_3$, 150 MHz) spectrum of compound **3** 16
Figure S29: DEPT 135 spectrum of compound **3** 17
Figure S30: HSQC spectrum of compound **3** 17
Figure S31: HMBC spectrum of compound **3** 18
Figure S32: COSY spectrum of compound **3**........ 18
Figure S33: ROESY spectrum of compound **3** 19
Figure S34: ESI-HR mass spectrum of compound **4** 19
Figure S35: IR spectrum of compound **4**........ 20
Figure S36: ^{1}H NMR ($CDCl_3$, 500 MHz) spectrum of compound **4** 20
Figure S37: ^{13}C NMR ($CDCl_3$, 125 MHz) spectrum of compound **4**........ 21
Figure S38: HSQC spectrum of compound **4** 21
Figure S39: HMBC spectrum of compound **4** 22
Figure S40: COSY spectrum of compound **4**........ 22
Figure S41: ROESY spectrum of compound **4** 23
Figure S42: ^{1}H NMR (CD_3OD, 500 MHz) spectrum of compound **5** 23
Figure S43: ^{1}H NMR ($CDCl_3$, 500 MHz) spectrum of compound **6** 24
Figure S44: ^{13}C NMR ($CDCl_3$, 125 MHz) spectrum of compound **6**........ 24
Figure S45: ^{1}H NMR ($CDCl_3$, 500 MHz) spectrum of a mixture of **7** and **8** 25

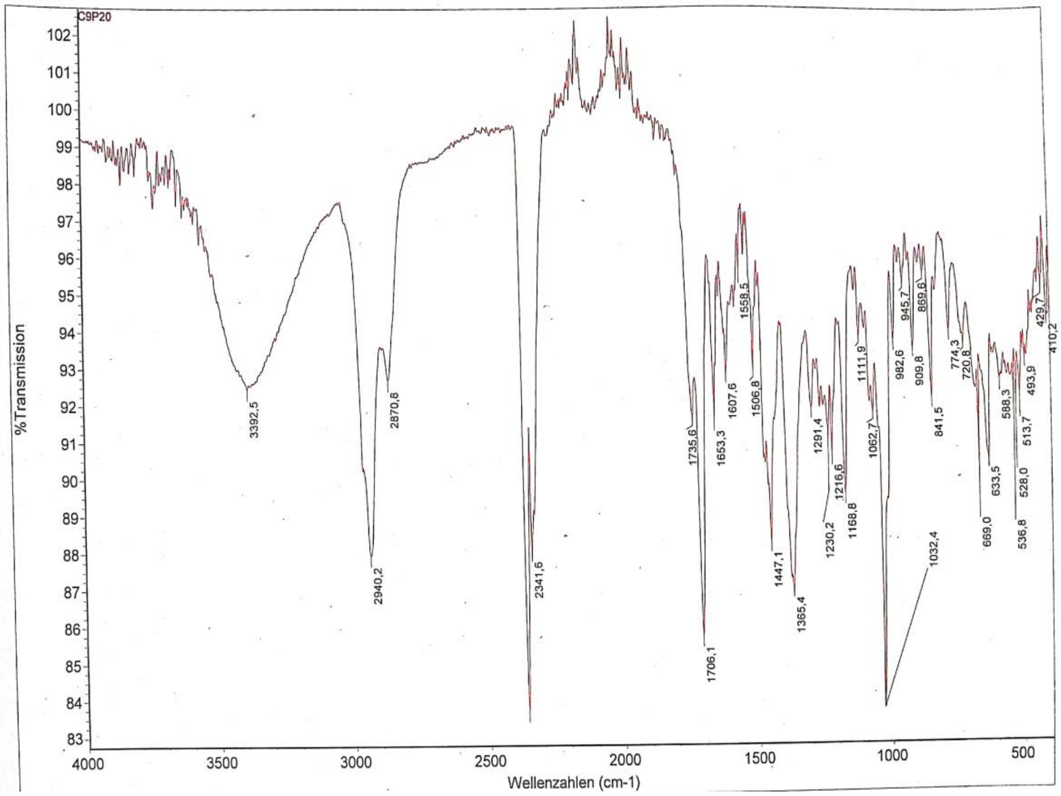

Figure S1: IR spectrum of compound **1**

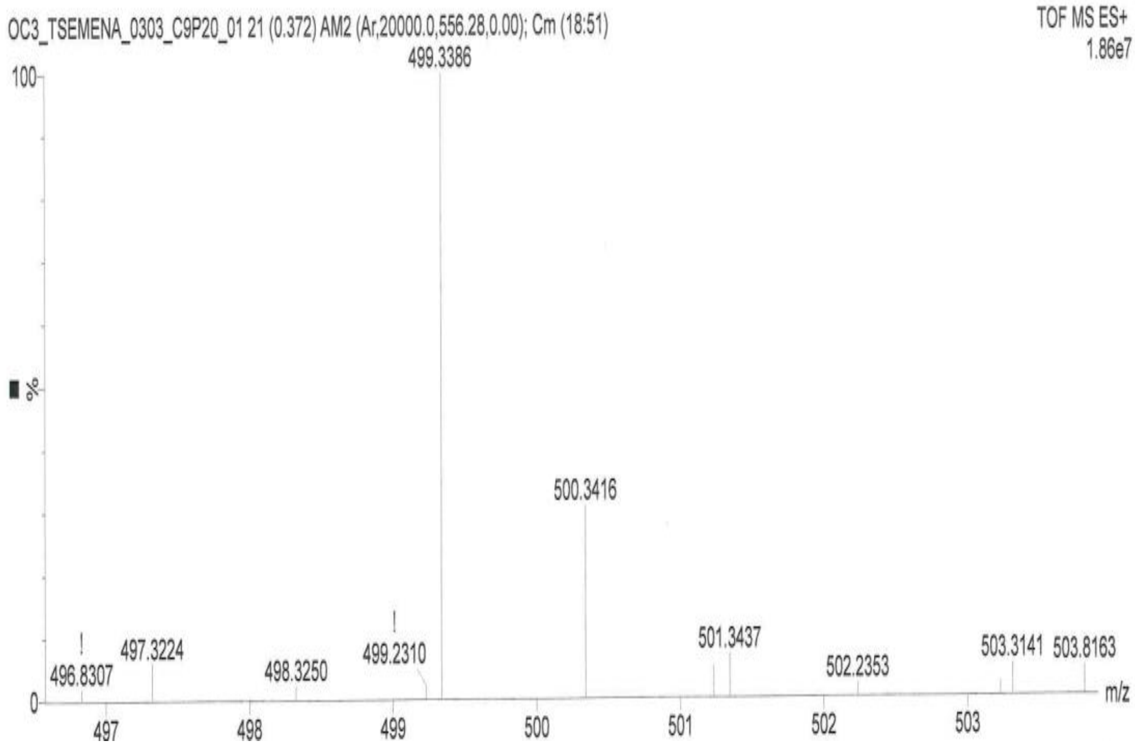

Figure S2: ESI-HR mass spectrum of compound **1**

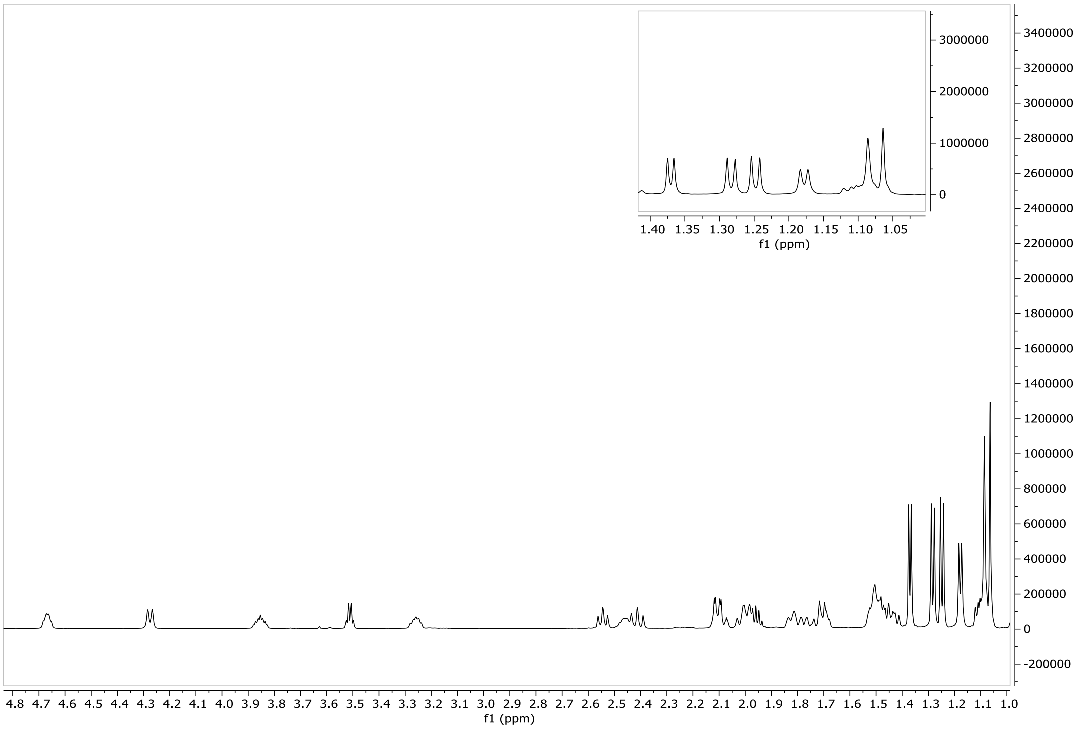

Figure S3: ^{1}H NMR ($CDCl_3$, 500 MHz) spectrum of compound **1**

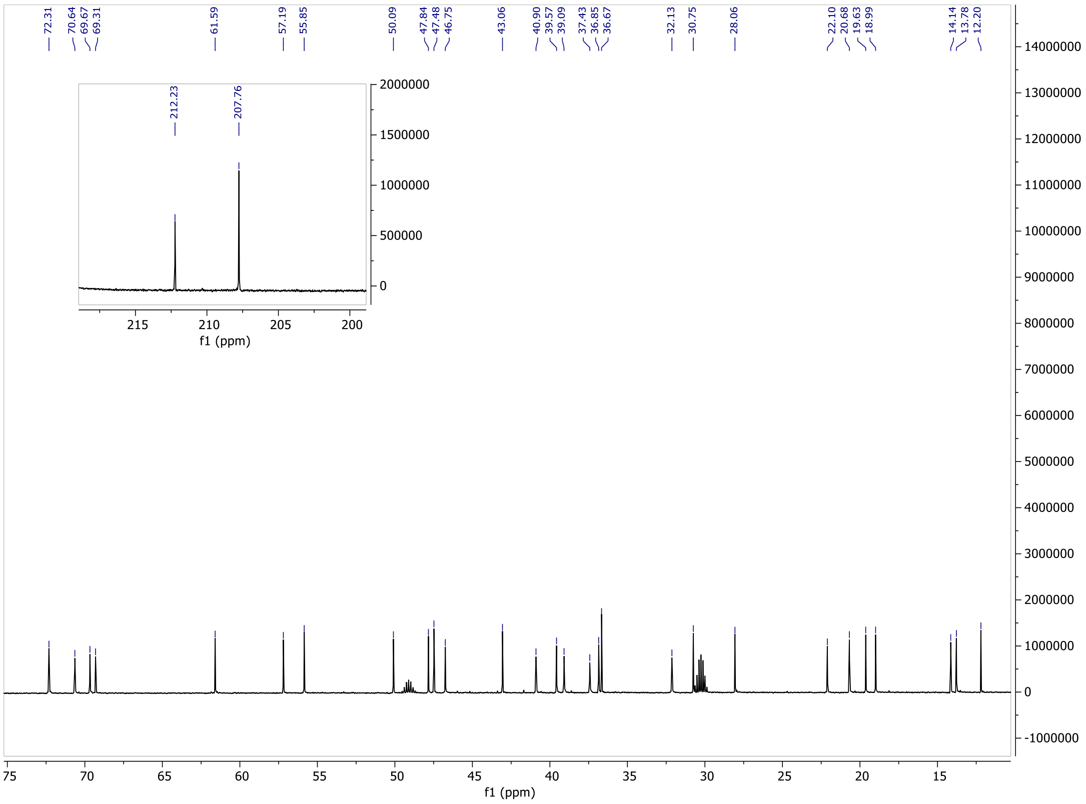

Figure S4: ^{13}C NMR ($CDCl_3$, 125 MHz) spectrum of compound **1**

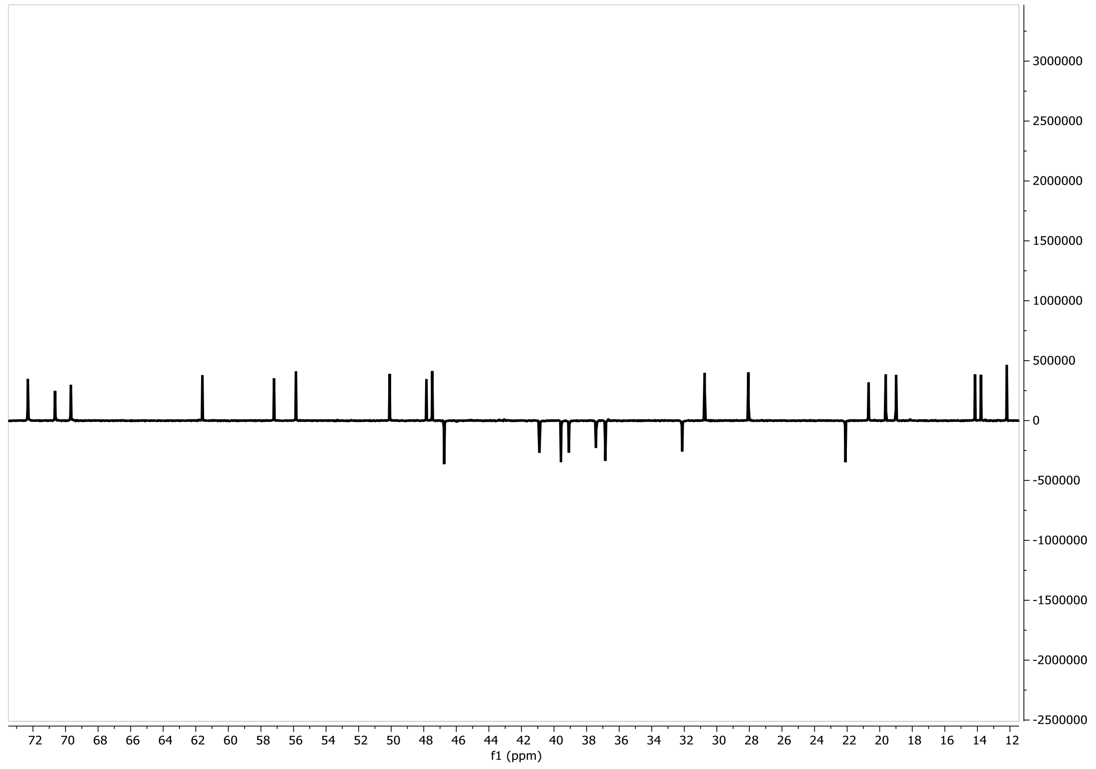

Figure S5: DEPT 135 spectrum of compound **1**

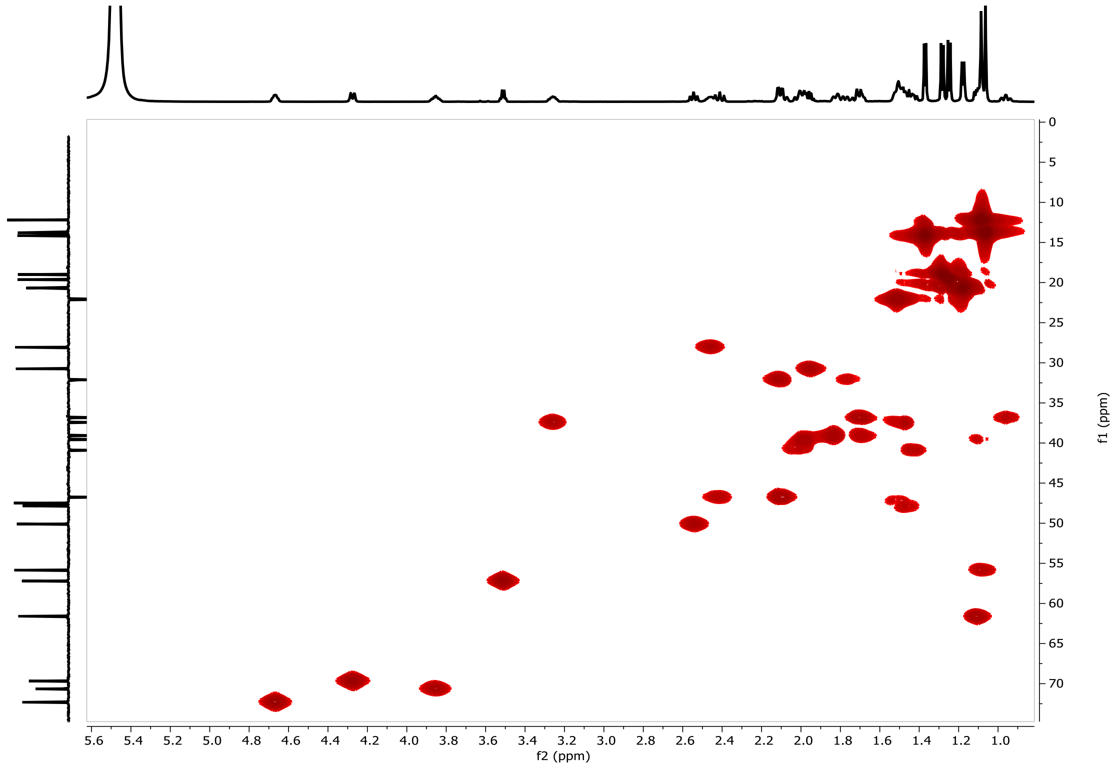

Figure S6: HSQC spectrum of compound **1**

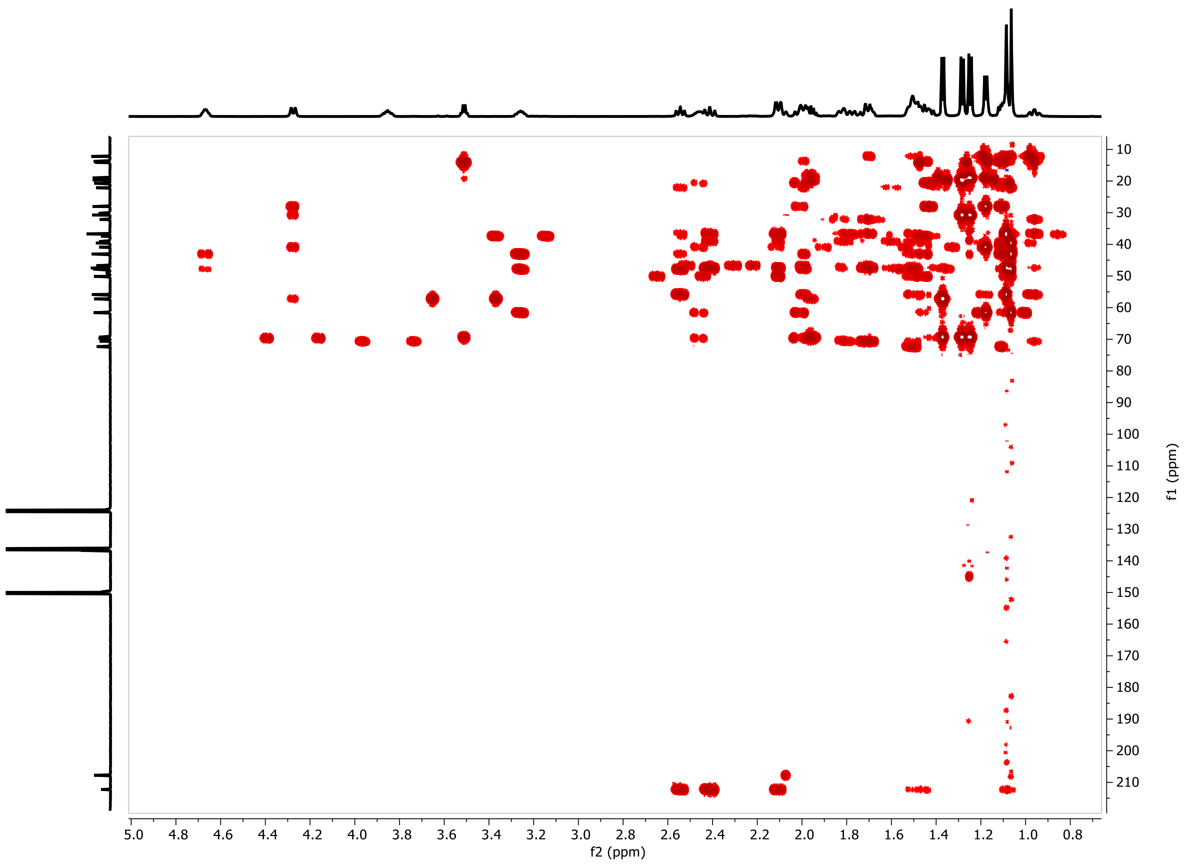

Figure S7: HMBC spectrum of compound **1**

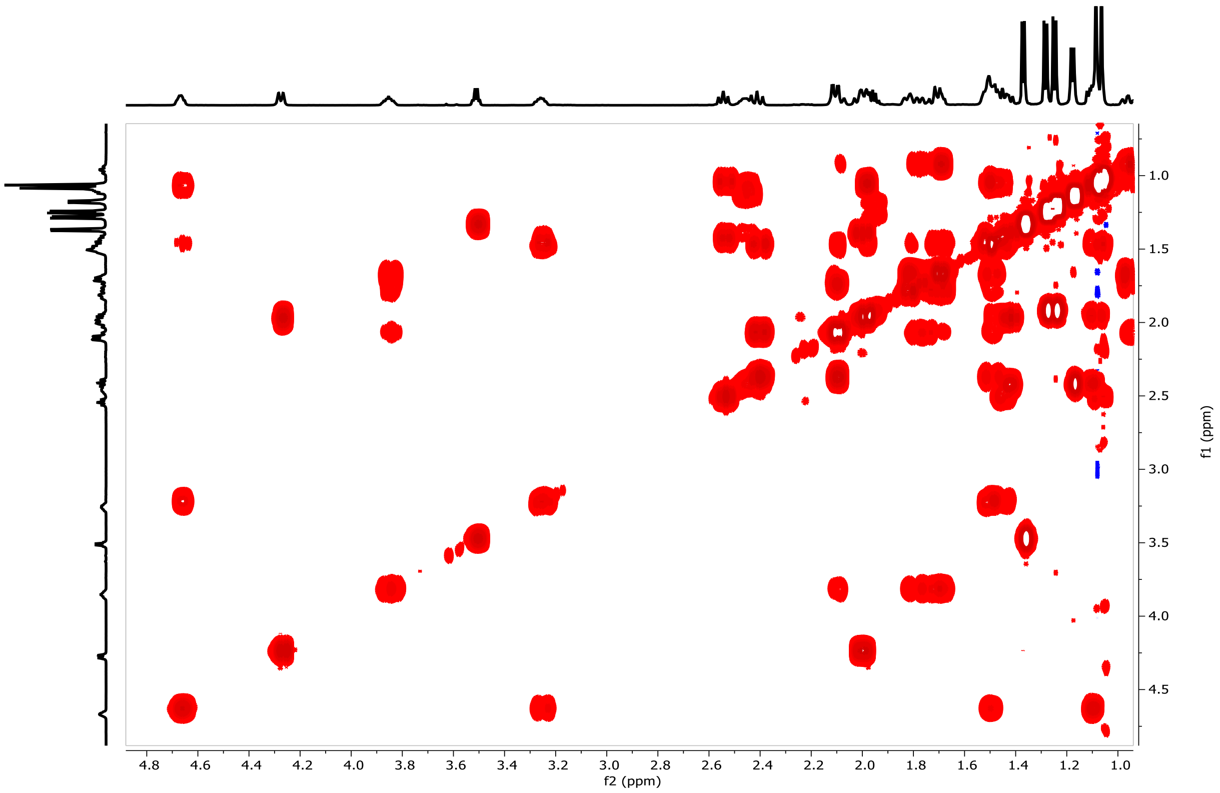

Figure S8: COSY spectrum of compound **1**

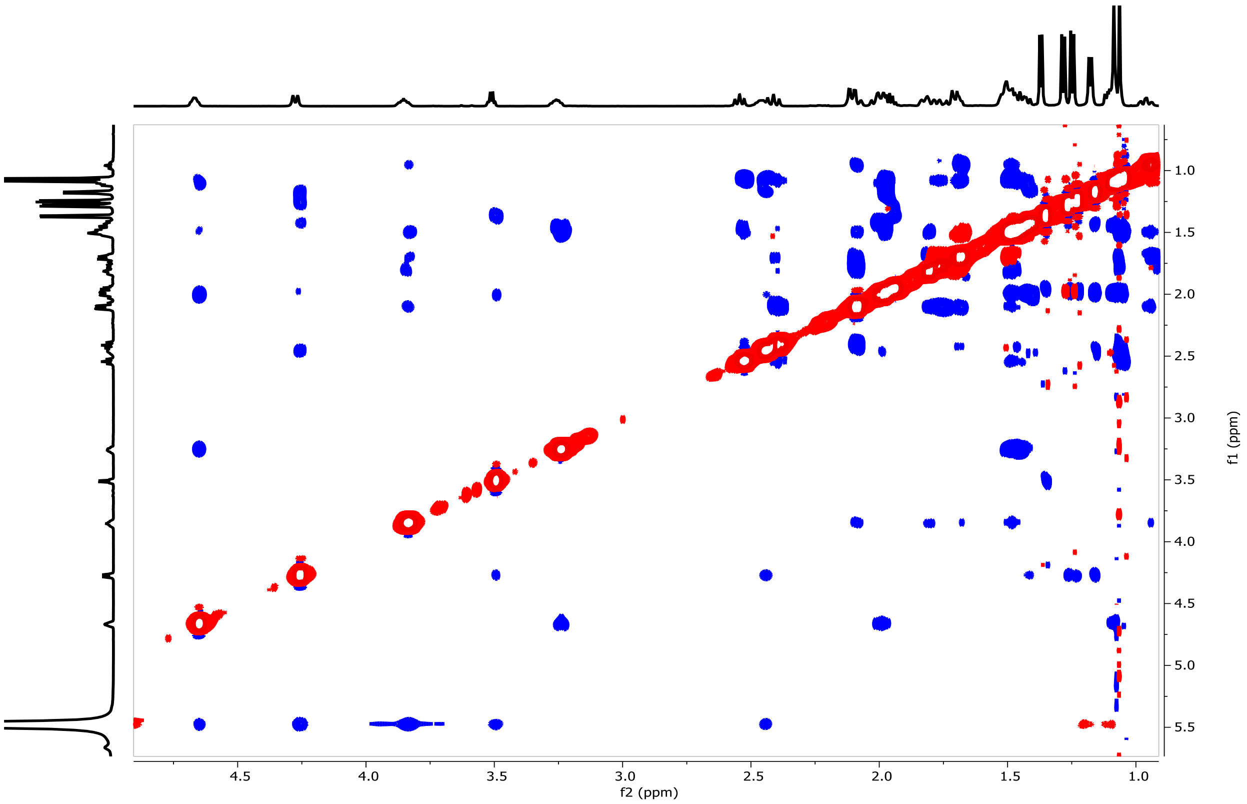

Figure S9: ROESY spectrum of compound **1**

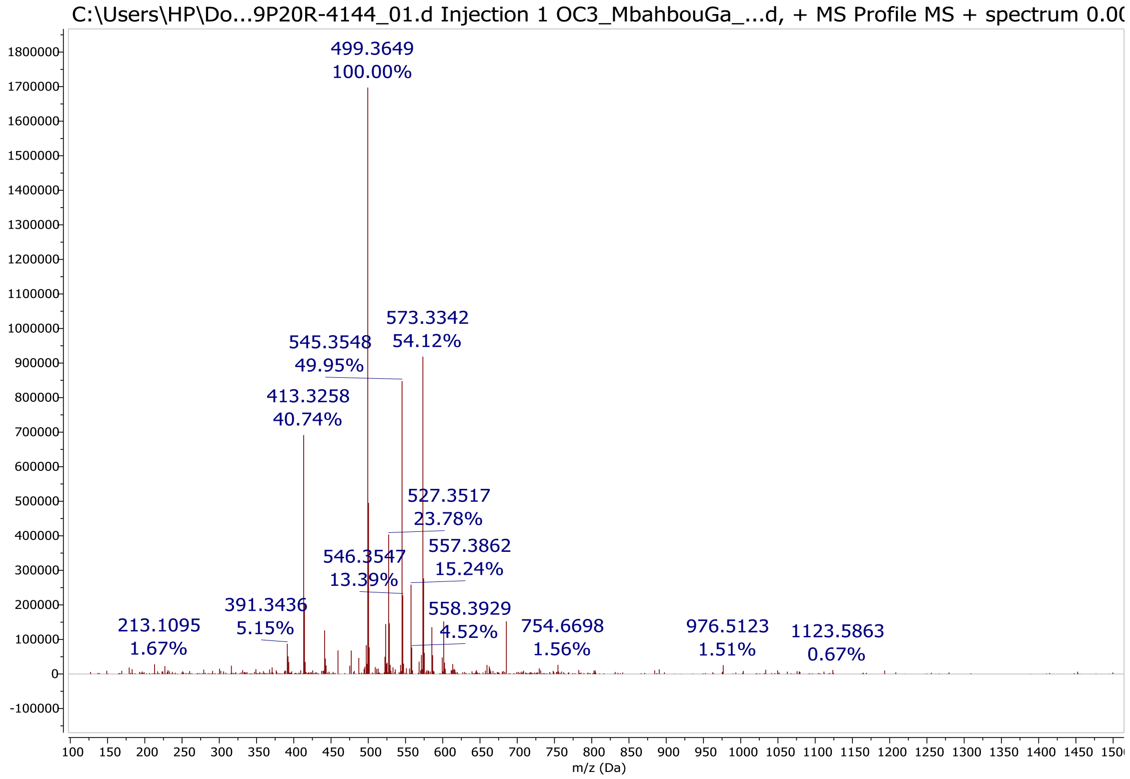

Figure S10: ESI-HR mass spectrum of compound **1a**

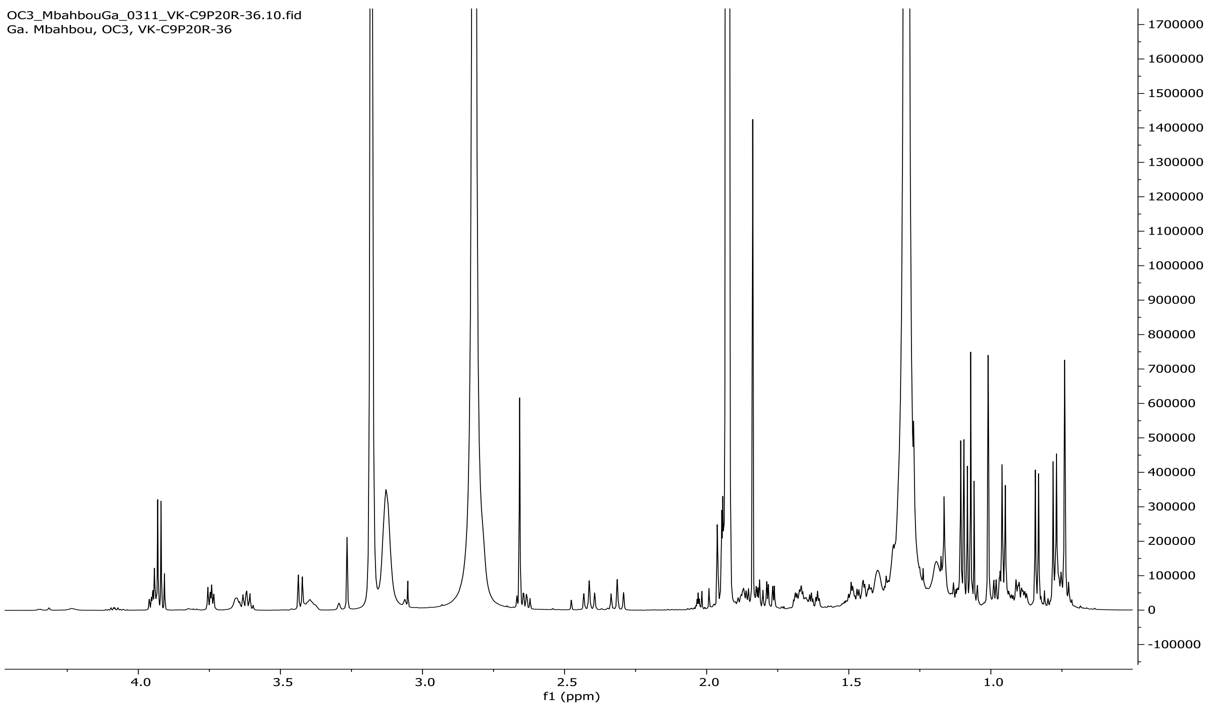

Figure S11: ^{1}H NMR (acetone-d_6, 600 MHz) spectrum of compound **1a**

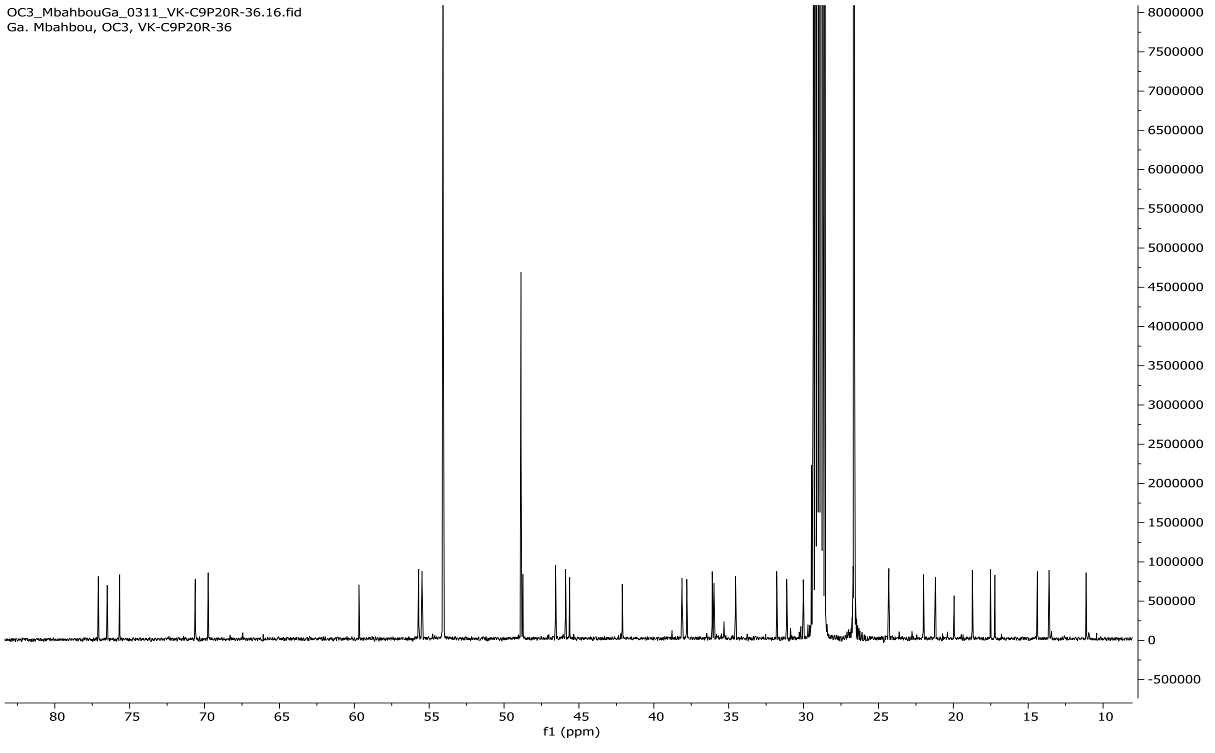

Figure S12: ^{13}C NMR (acetone-d_6, 150 MHz) spectrum of compound **1a**

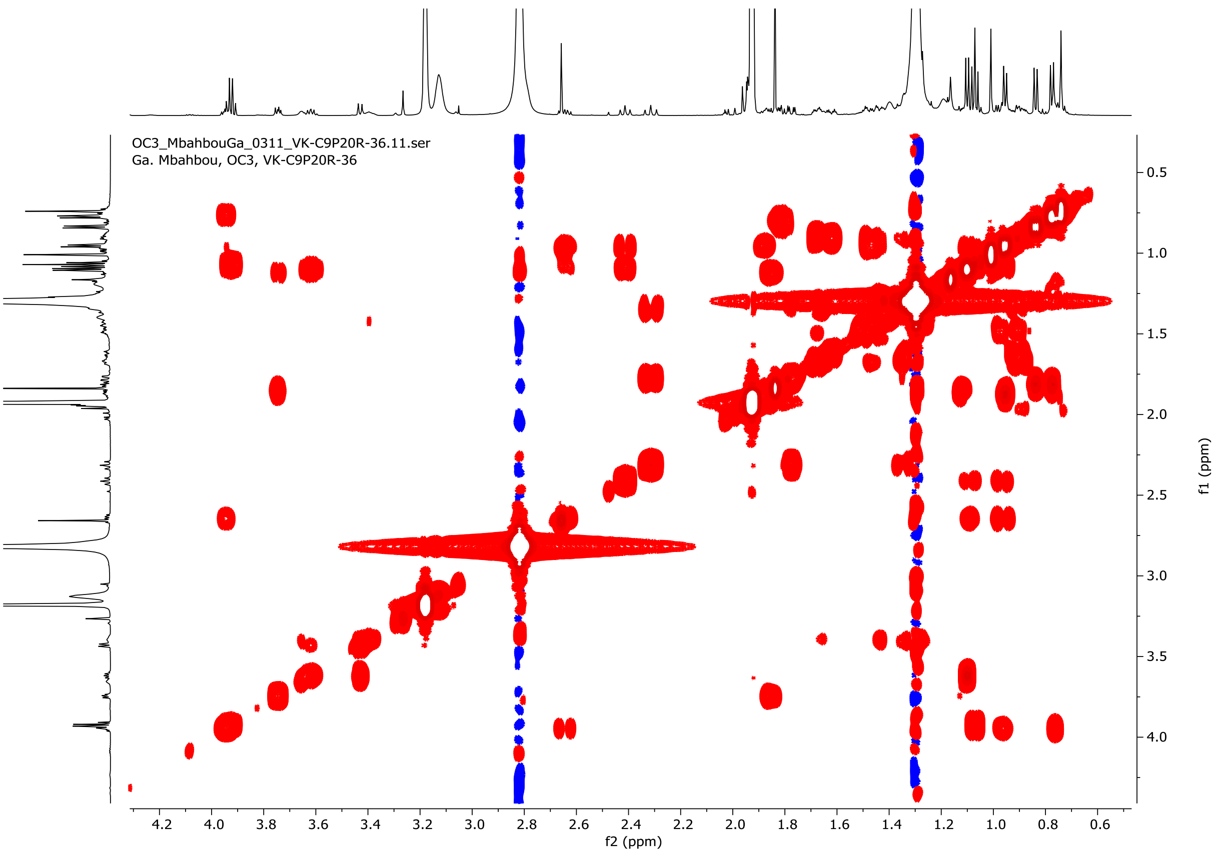

Figure S13: COSY spectrum of compound **1a**

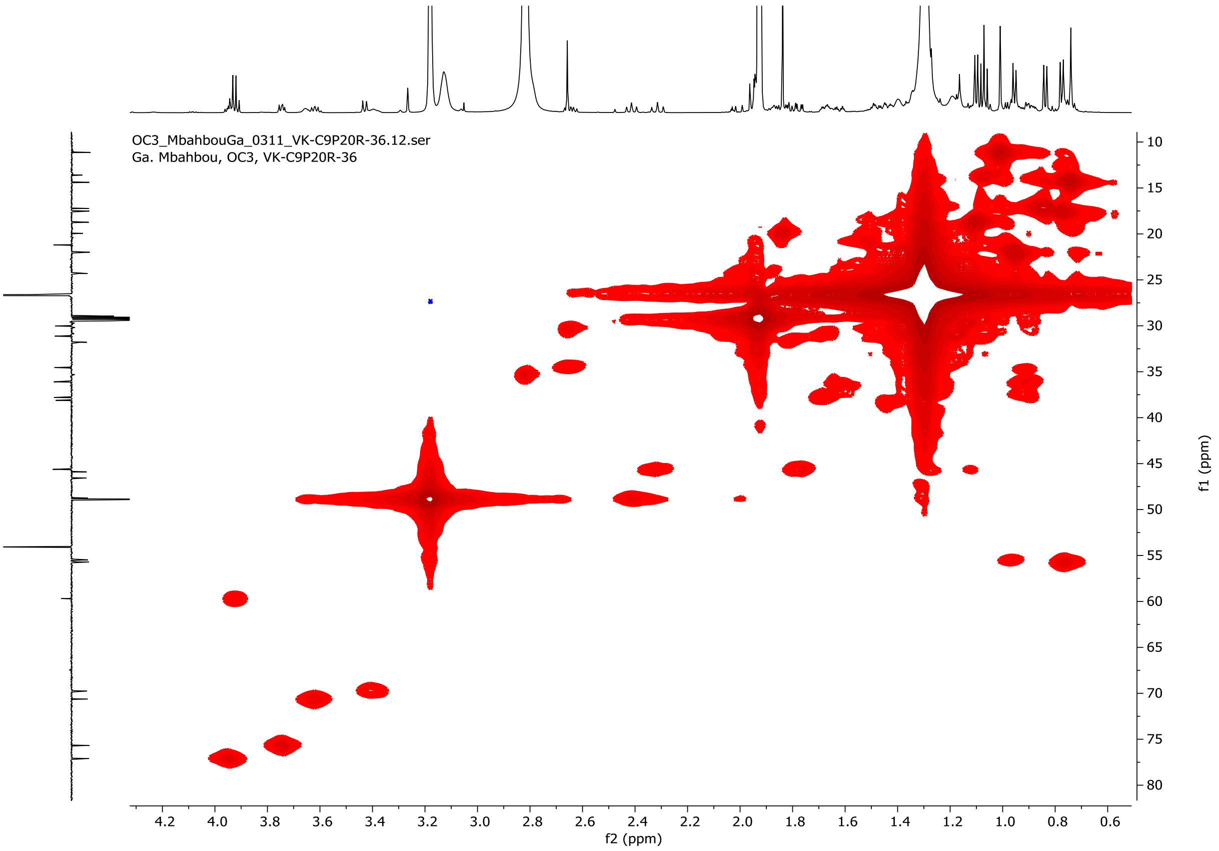

Figure S14: HSQC spectrum of compound **1a**

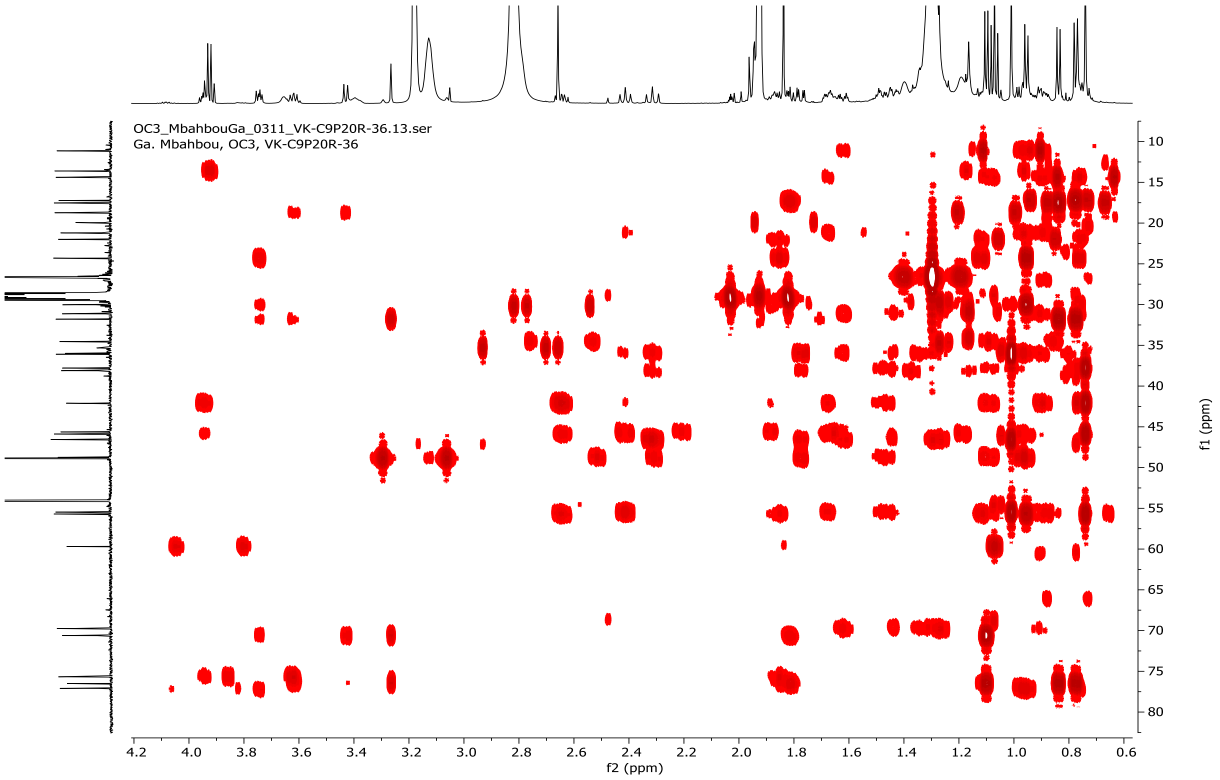

Figure S15: HMBC spectrum of compound **1a**

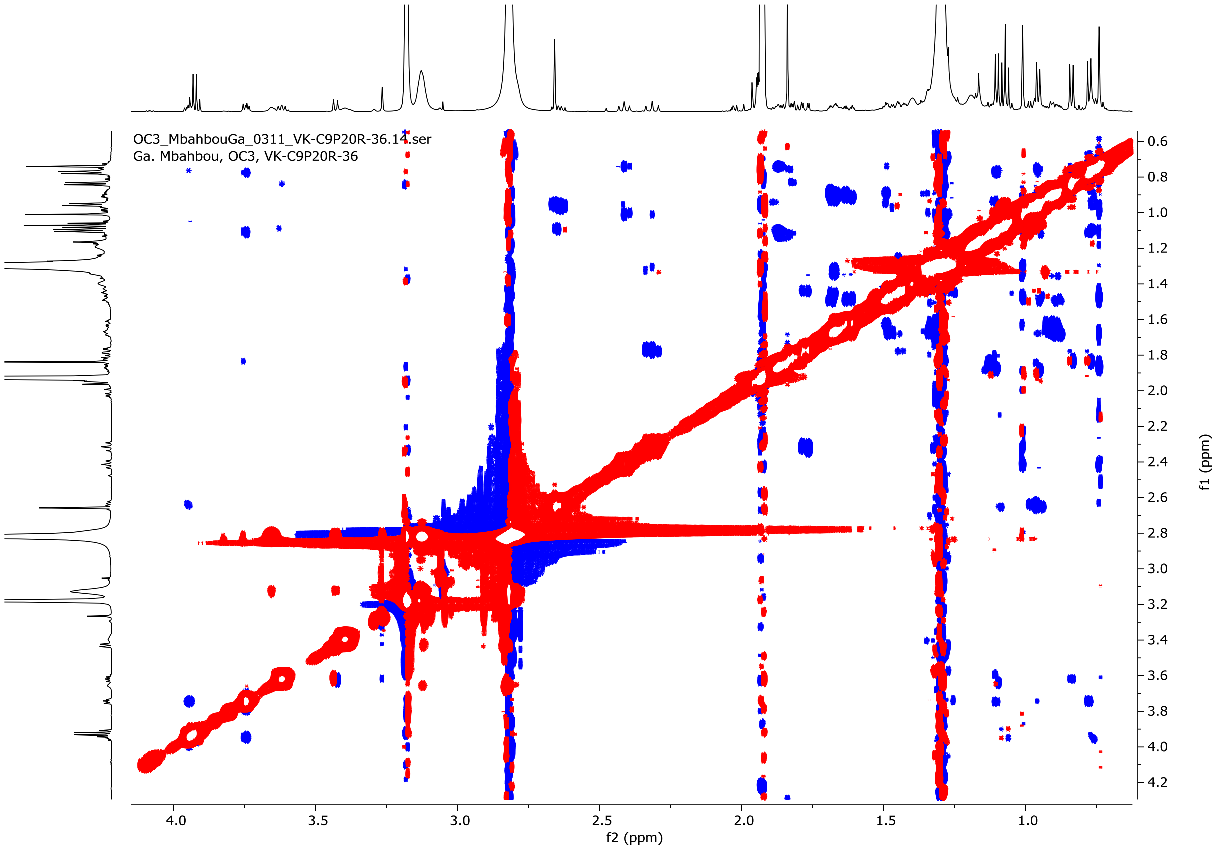

Figure S16: NOESY spectrum of **1a**

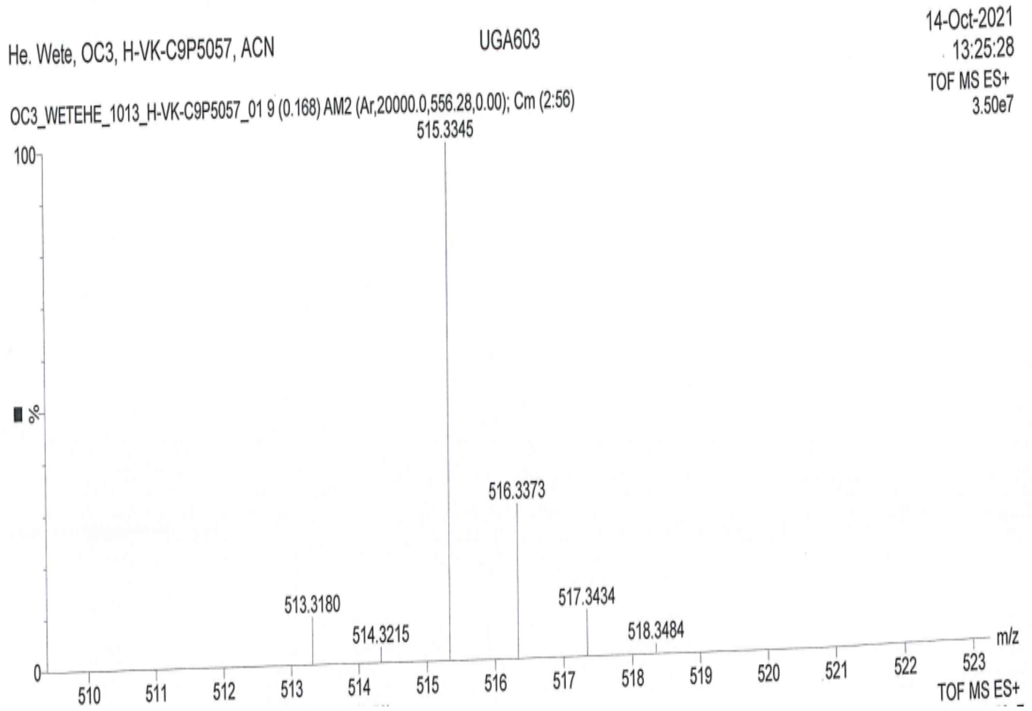

Figure S17: ESI-HR mass spectrum of compound **2**

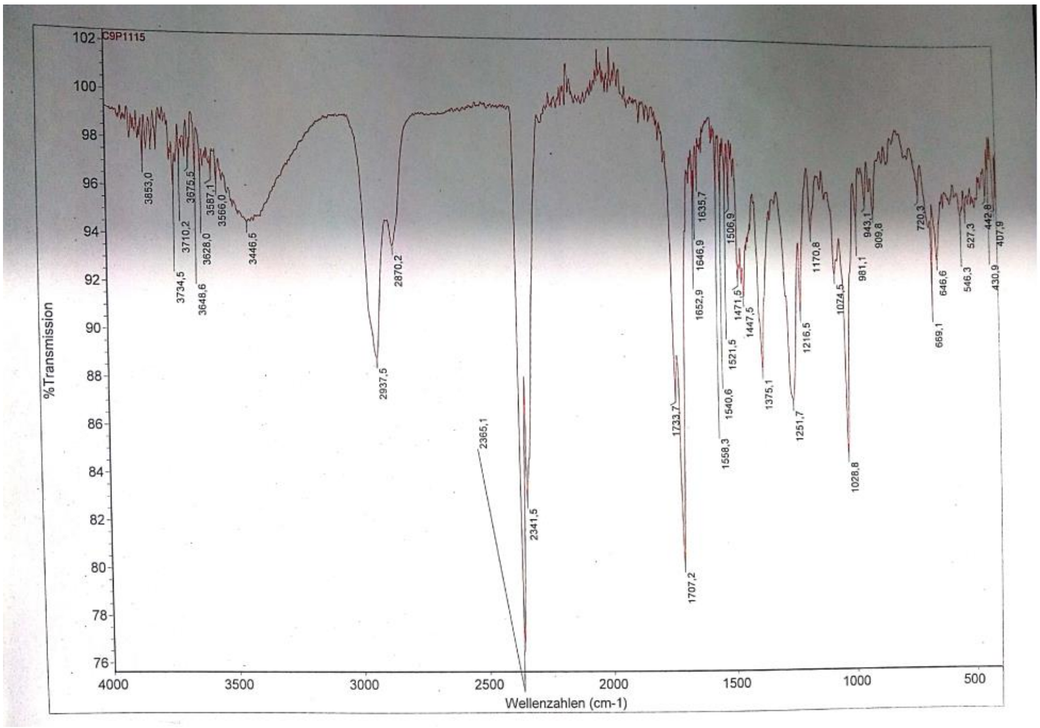

Figure S18: IR spectrum of compound **2**

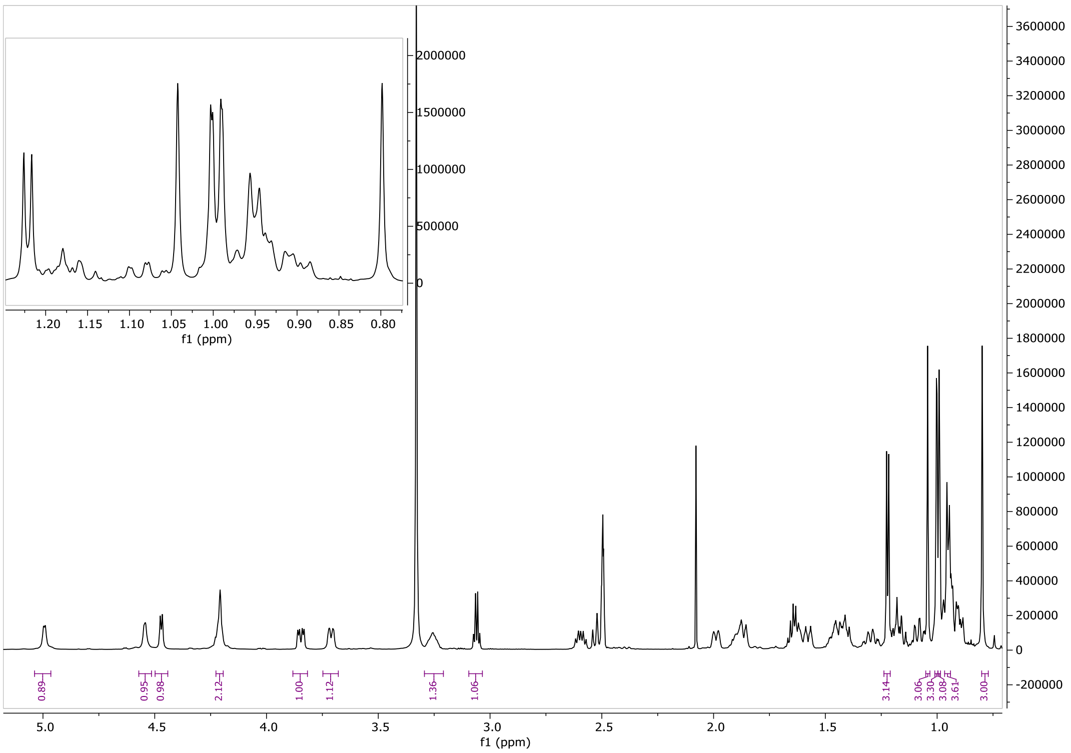

Figure S19: ^{1}H NMR ($CDCl_3$, 500 MHz) spectrum of compound **2**

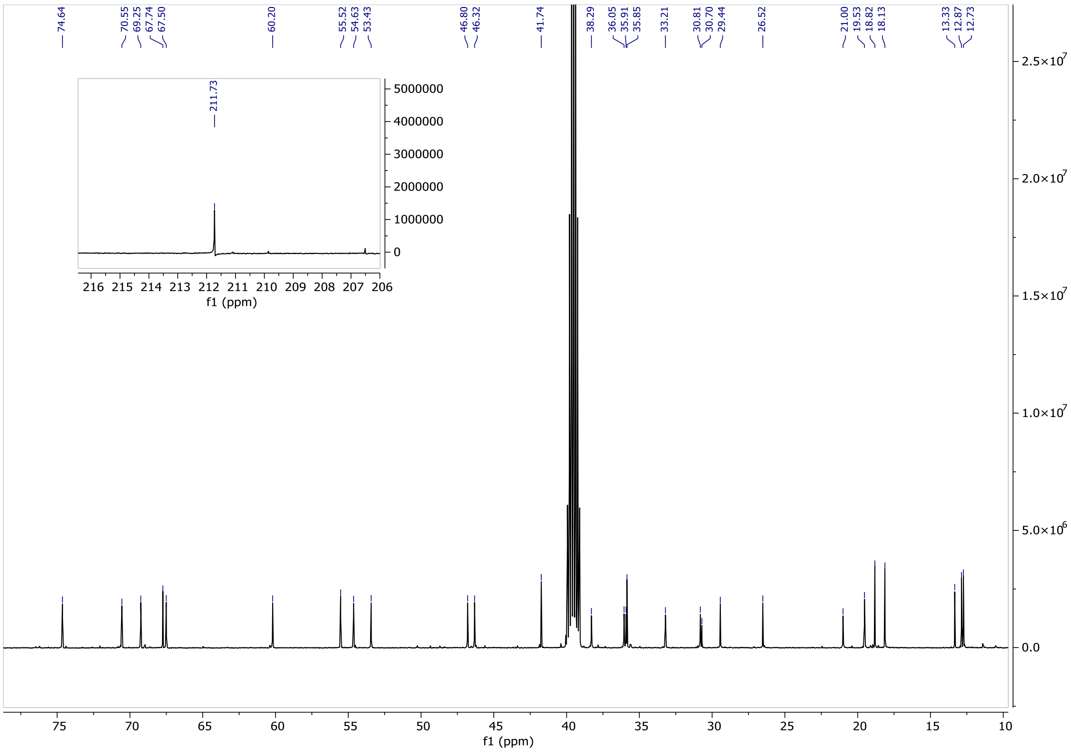

Figure S20: ^{13}C NMR ($CDCl_3$, 125 MHz) spectrum of compound **2**

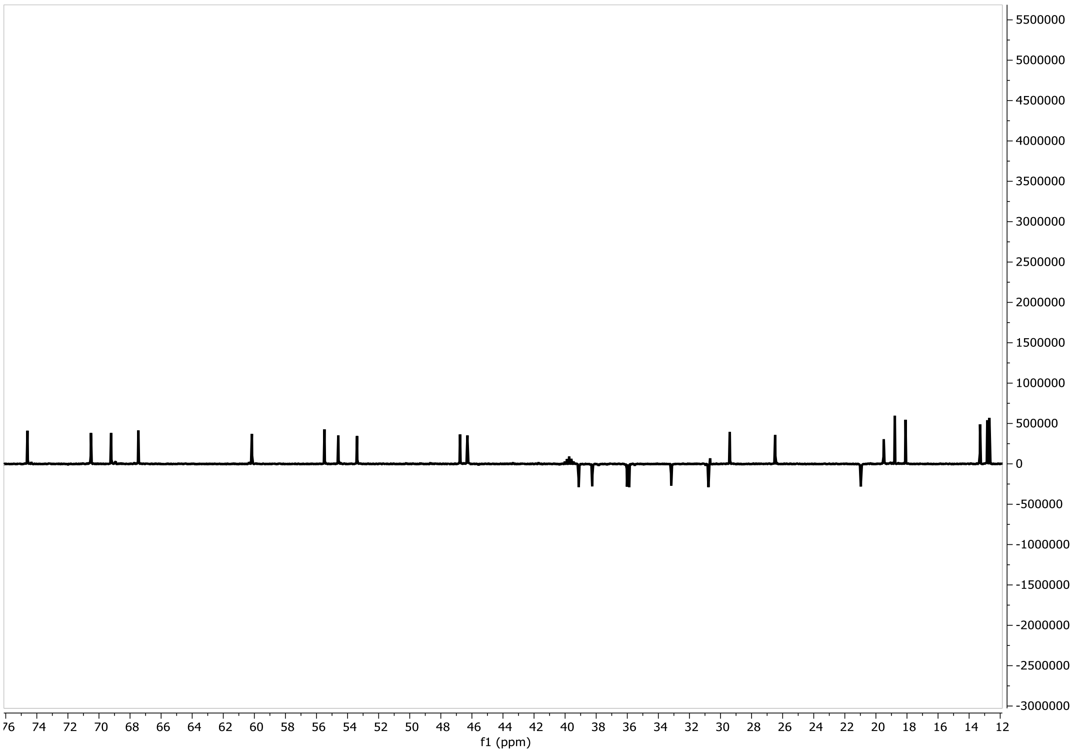

Figure S21: DEPT 135 spectrum of compound **2**

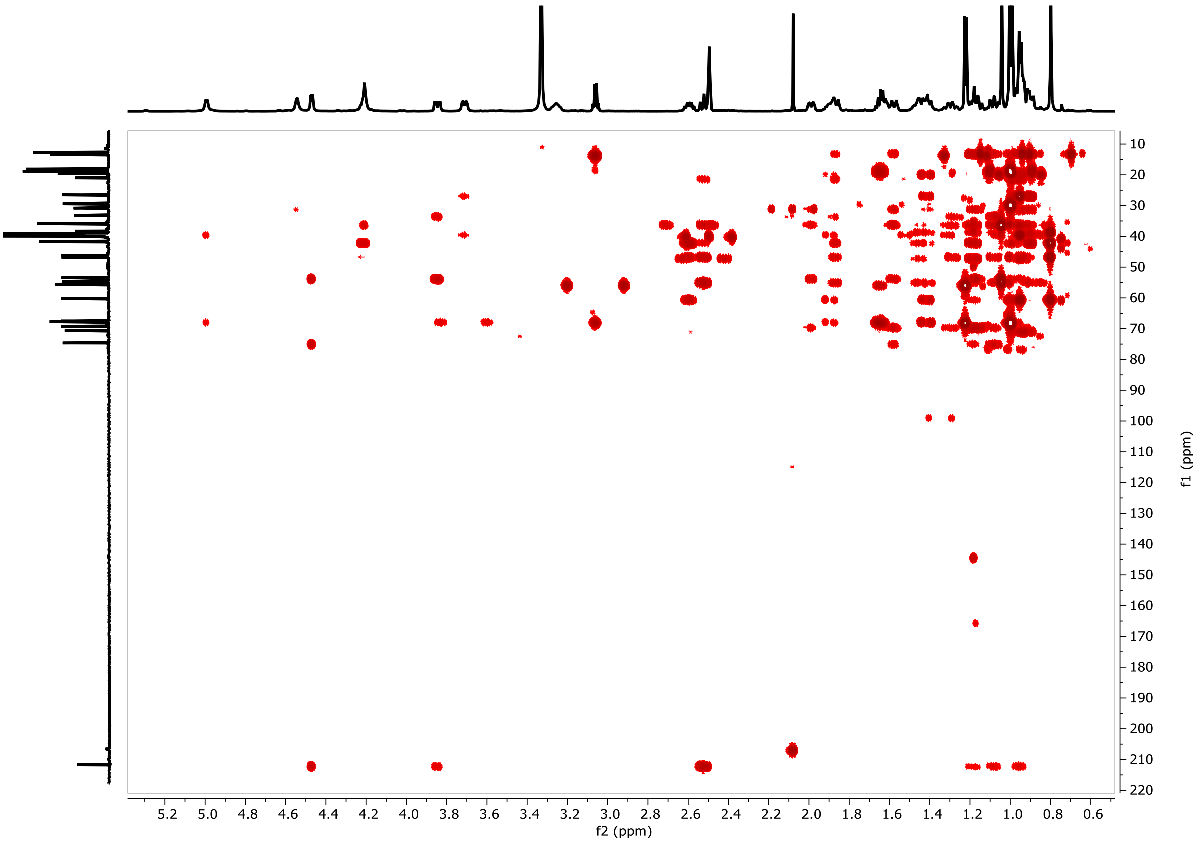

Figure S22: HMBC spectrum of compound **2**

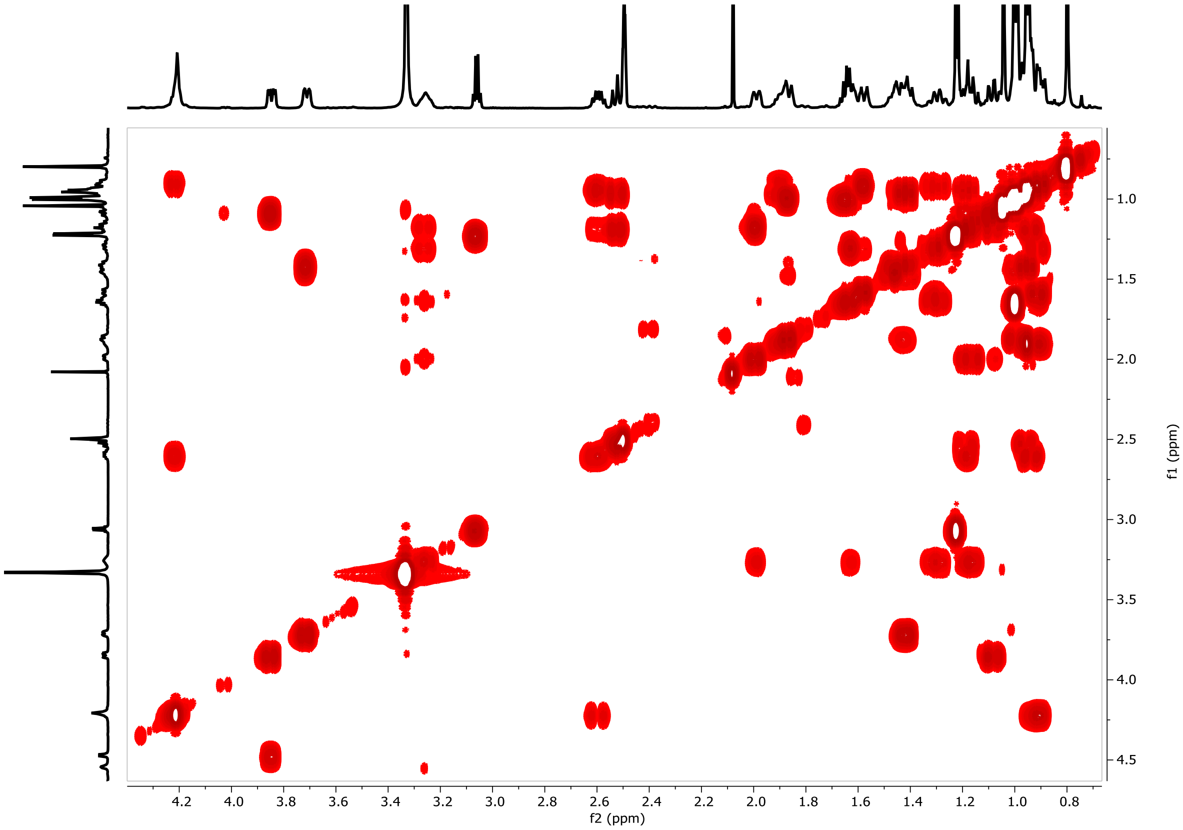

Figure S23: COSY spectrum of compound **2**

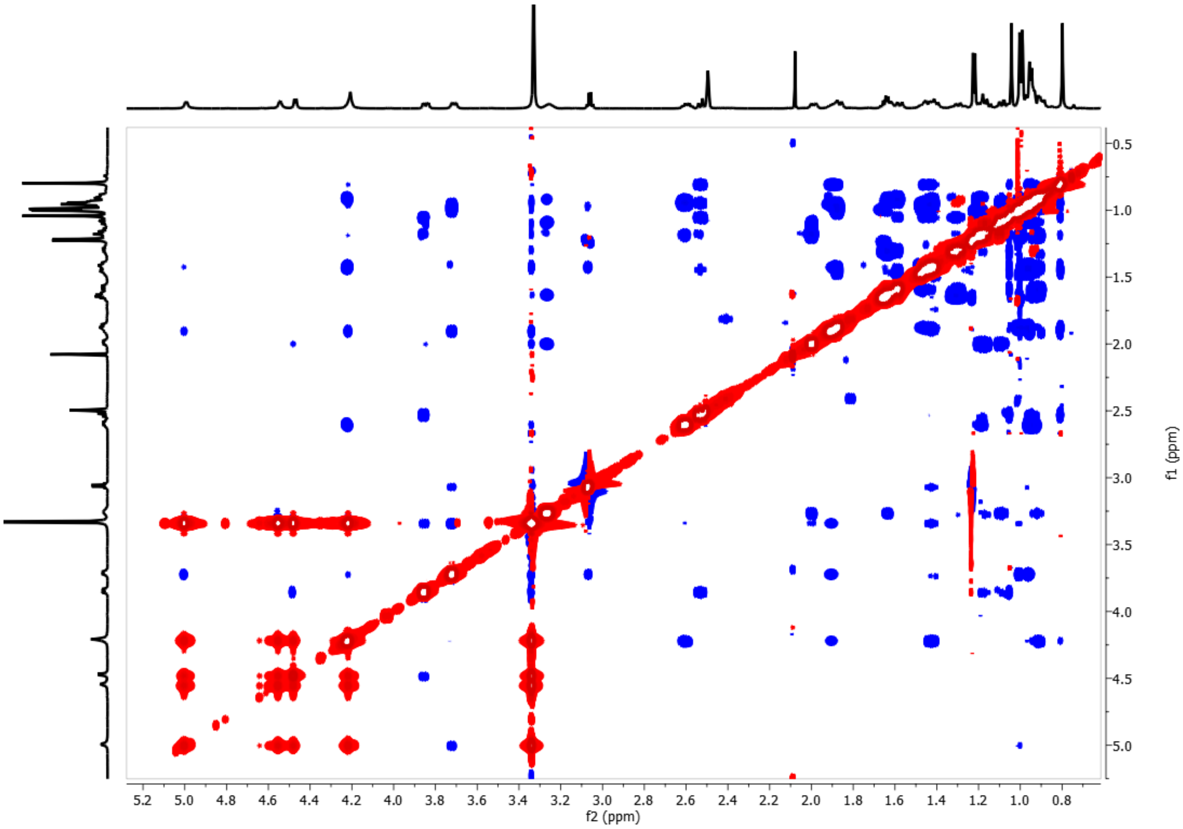

Figure S24: ROESY spectrum of compound **2**

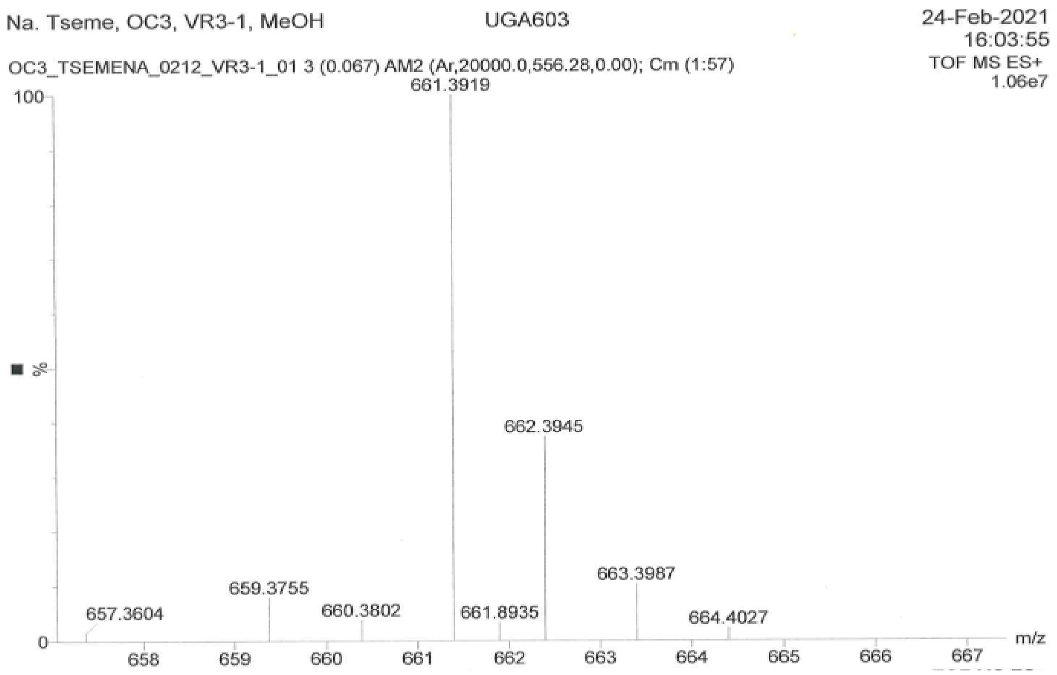

Figure S25: ESI-HR mass spectrum of compound **3**

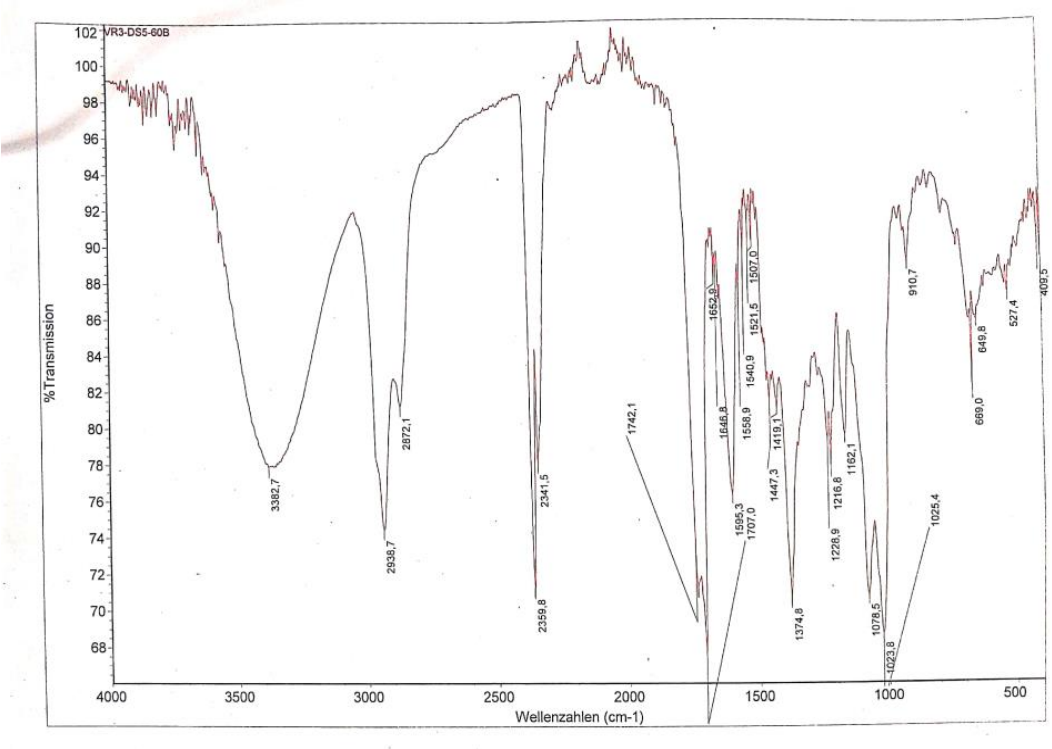

Figure S26: IR spectrum of compound **3**

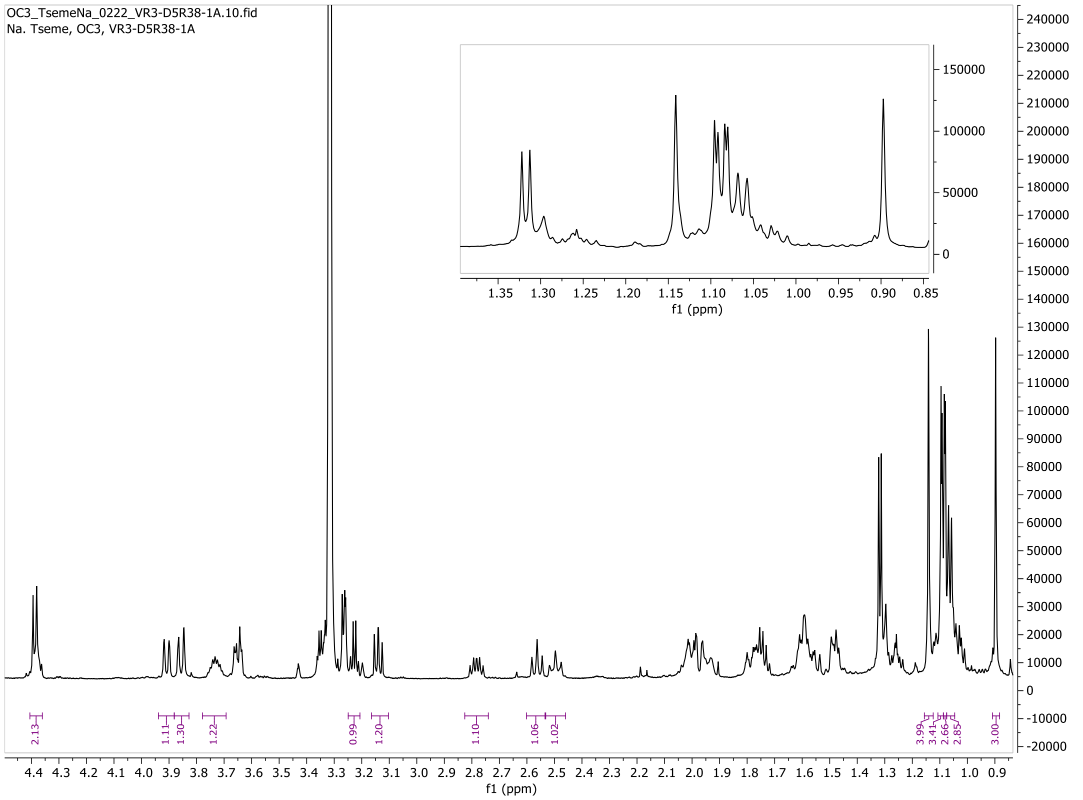

Figure S27: ^{1}H NMR ($CDCl_3$, 600 MHz) spectrum of compound **3**

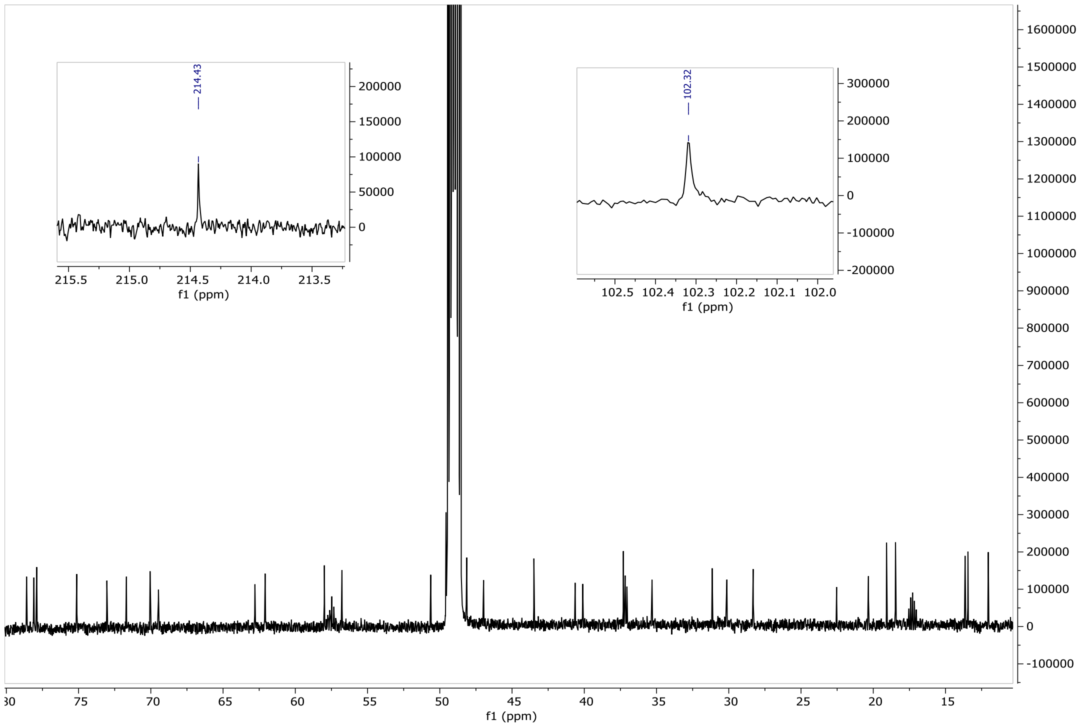

Figure S28: ^{13}C ($CDCl_3$, 150 MHz) spectrum of compound **3**

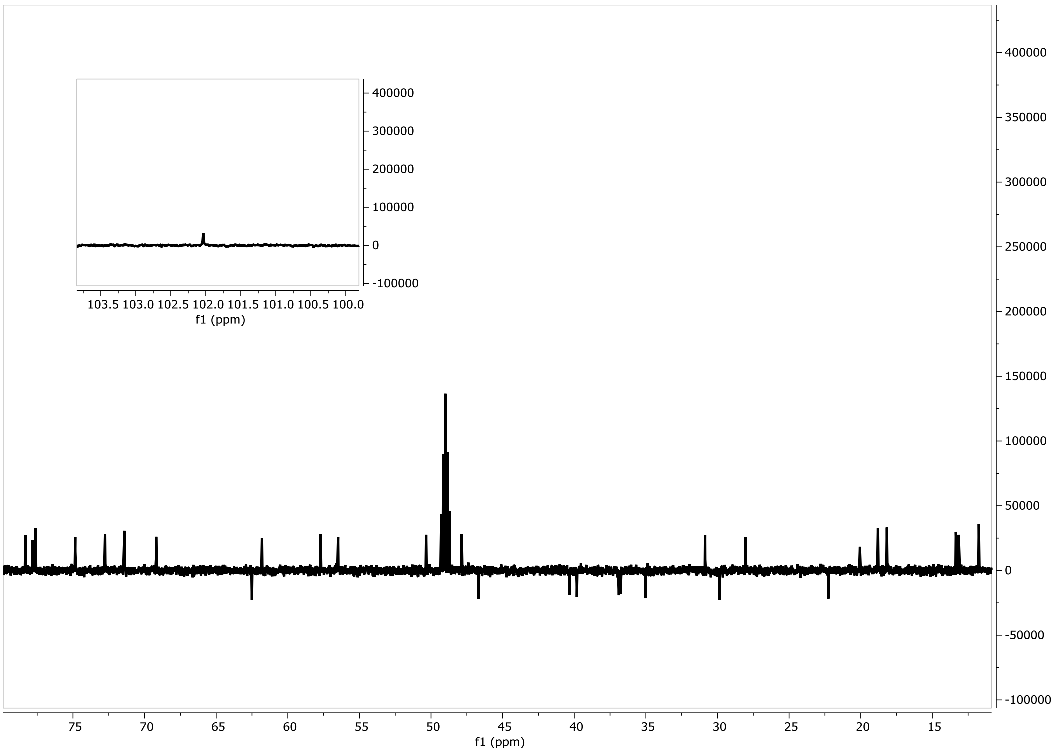

Figure S29: DEPT 135 spectrum of compound **3**

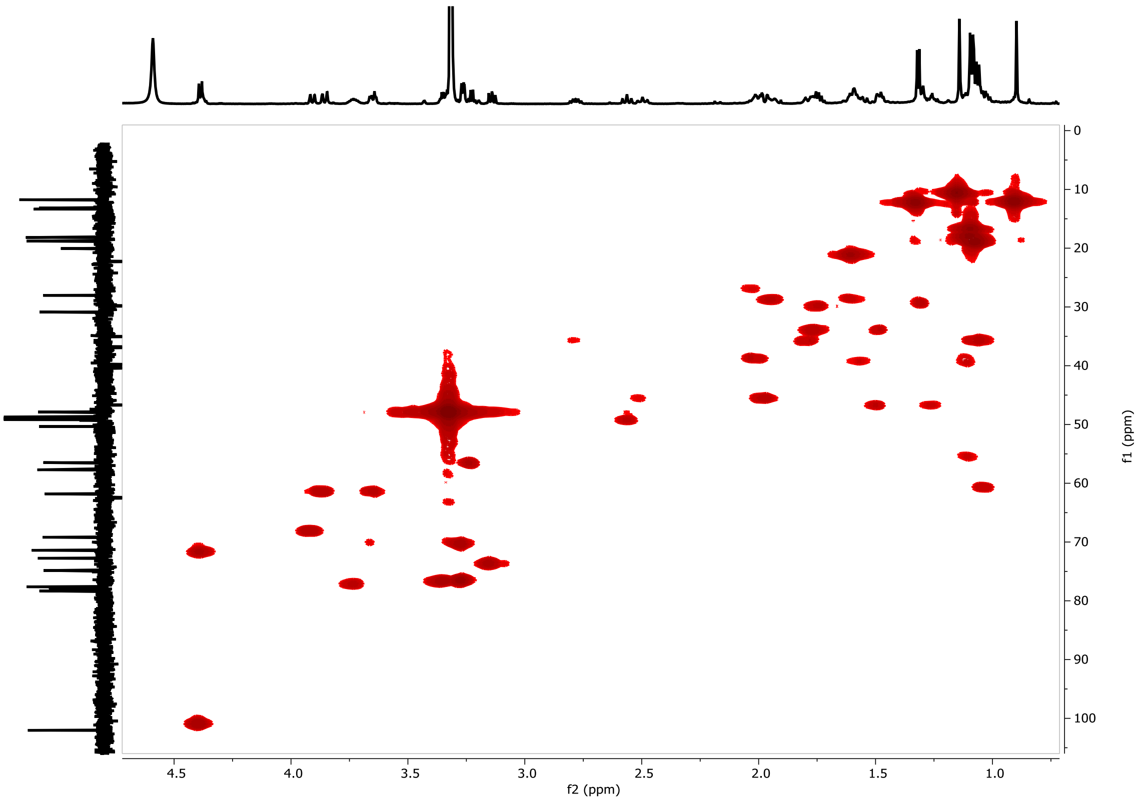

Figure S30: HSQC spectrum of compound **3**

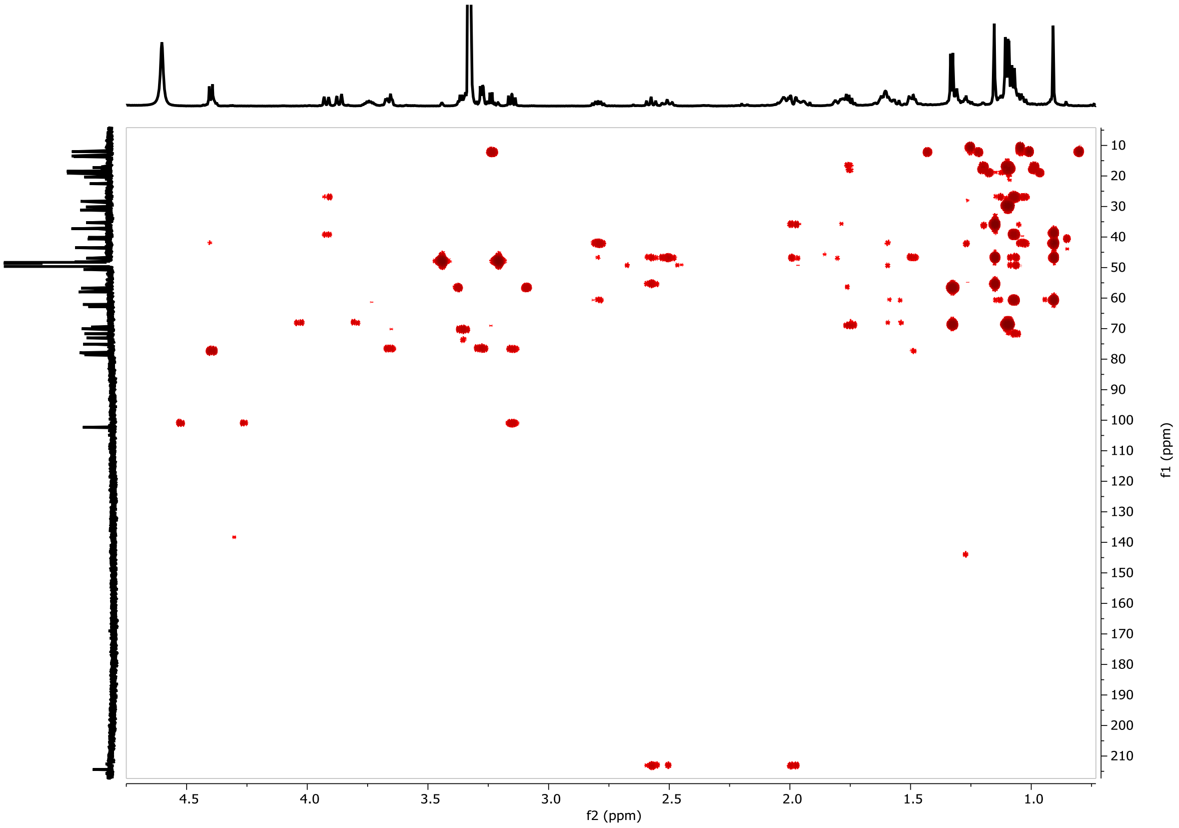

Figure S31: HMBC spectrum of compound **3**

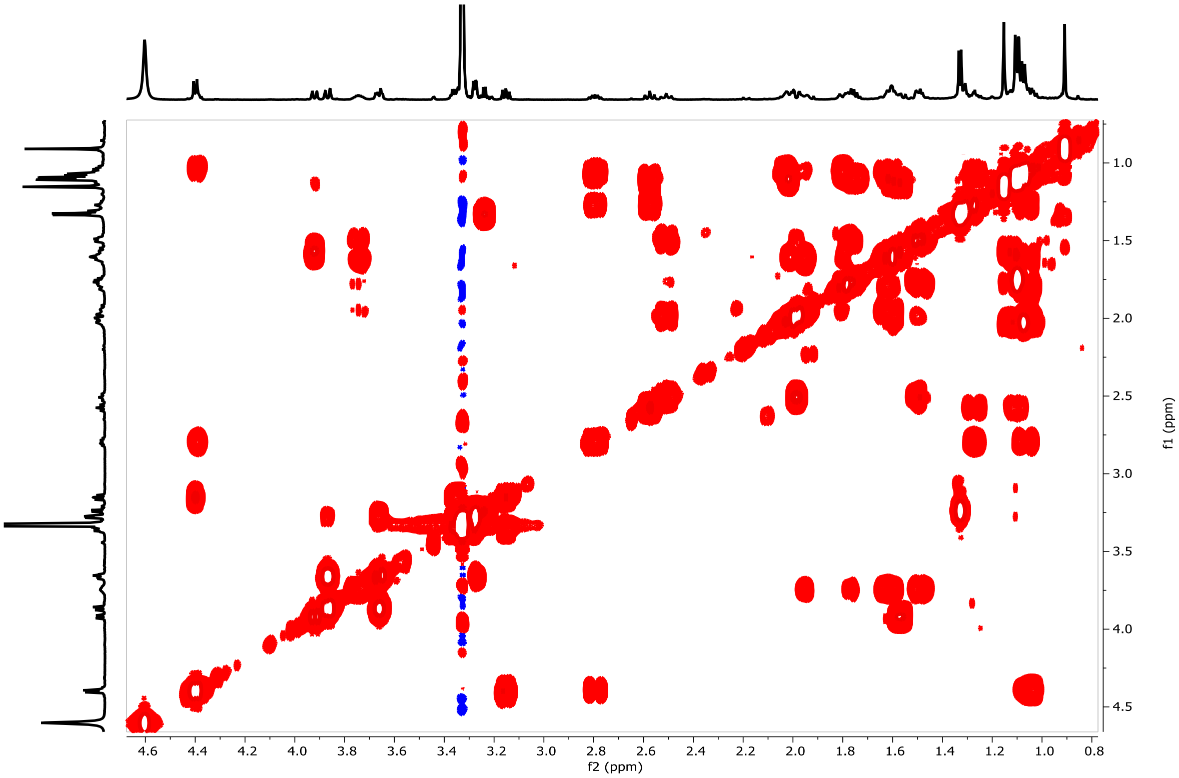

Figure S32: COSY spectrum of compound **3**

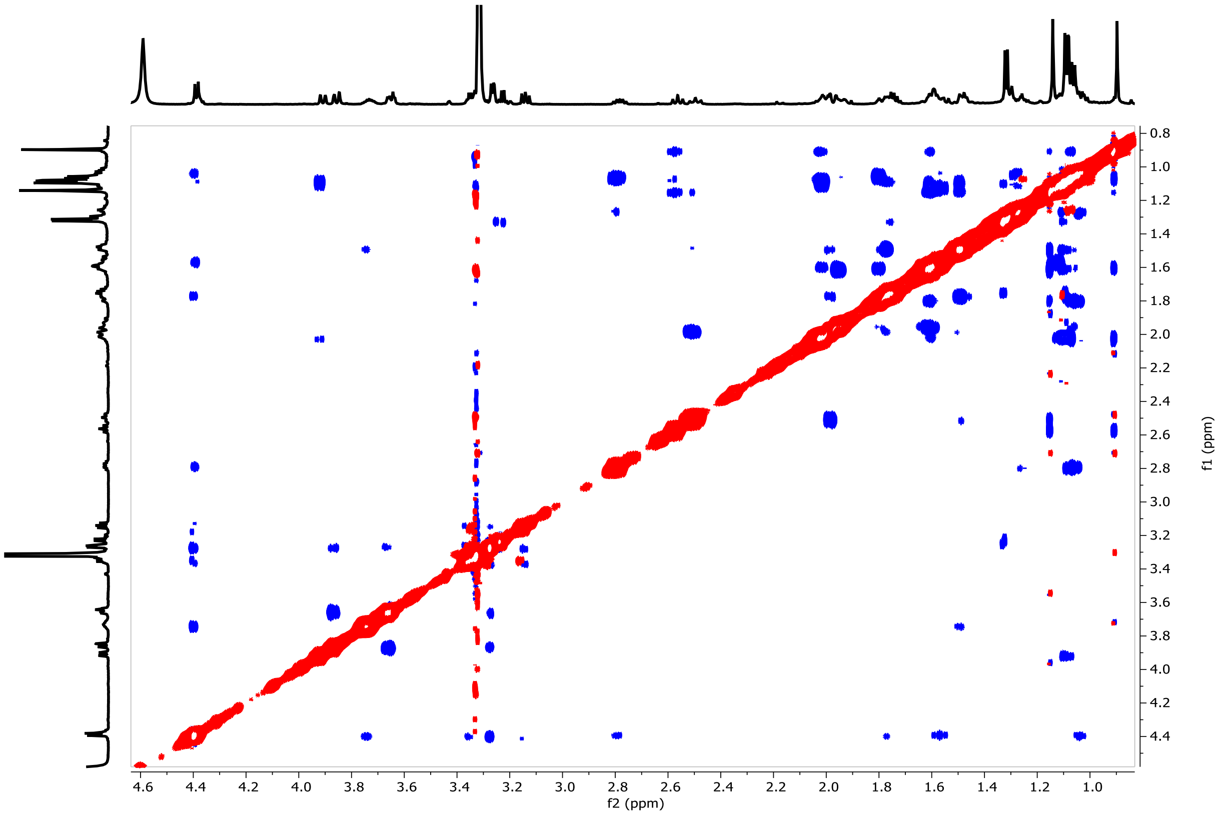

Figure S33: ROESY spectrum of compound **3**

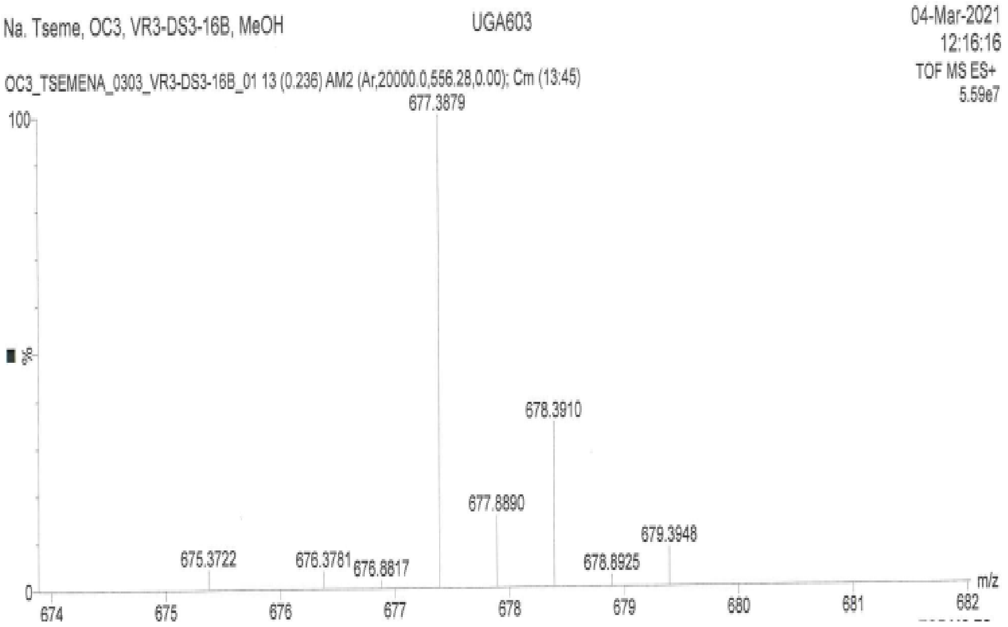

Figure S34: ESI-HR mass spectrum of compound **4**

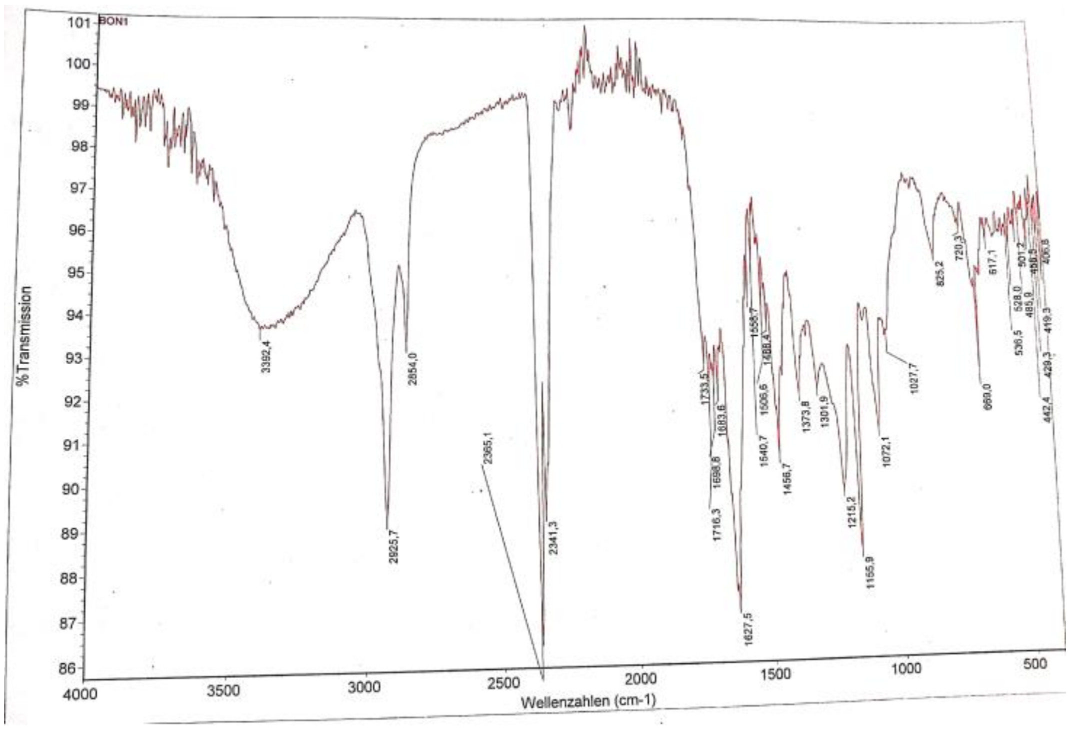

Figure S35: IR spectrum of compound **4**

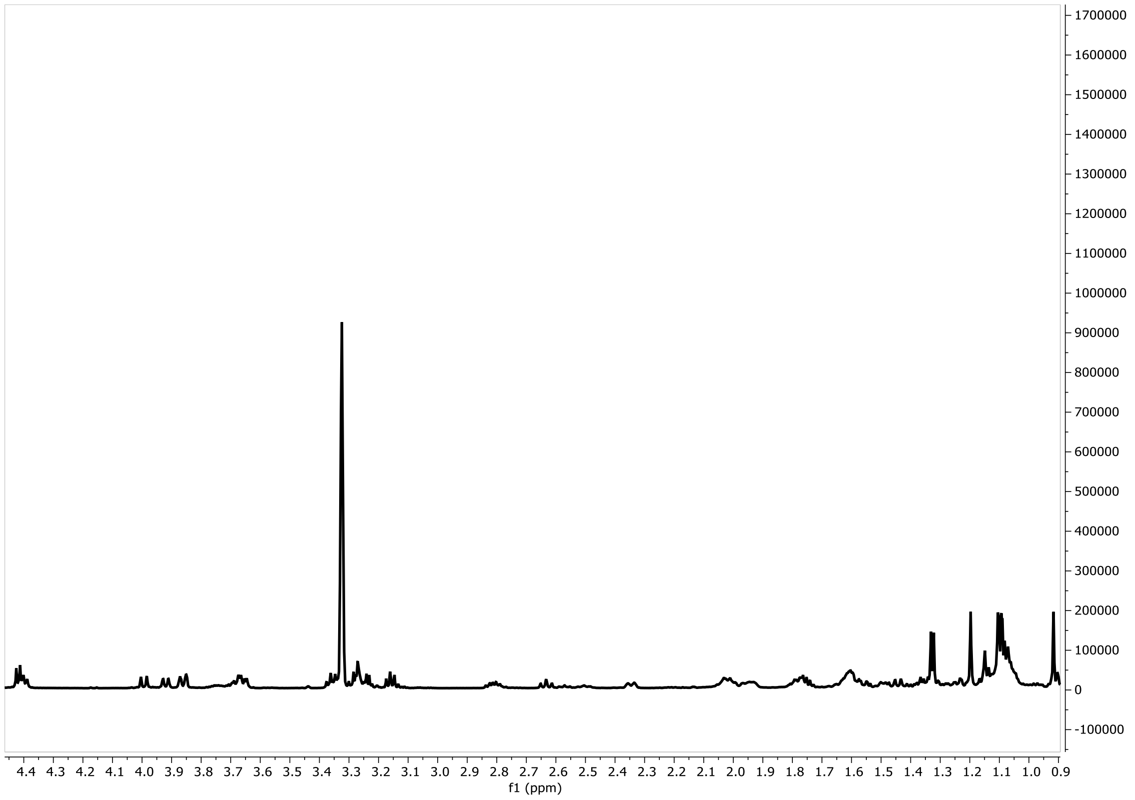

Figure S36: ^{1}H NMR ($CDCl_3$, 500 MHz) spectrum of compound **4**

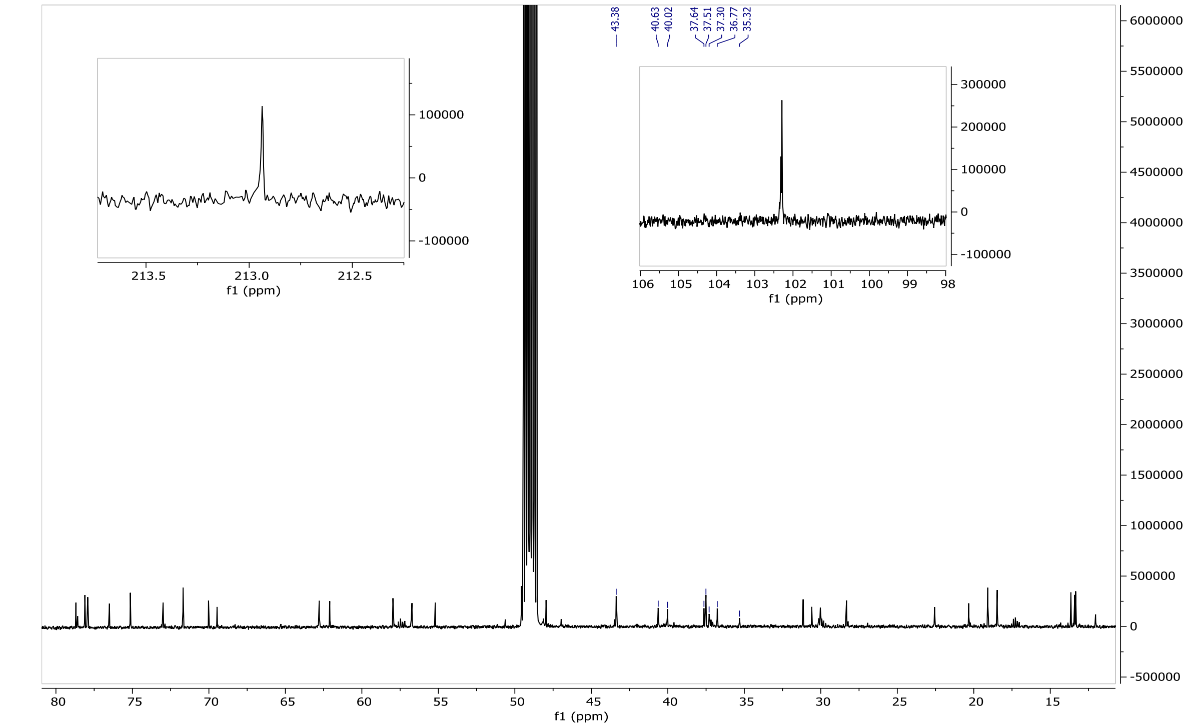

Figure S37: ^{13}C NMR ($CDCl_3$, 125 MHz) spectrum of compound **4**

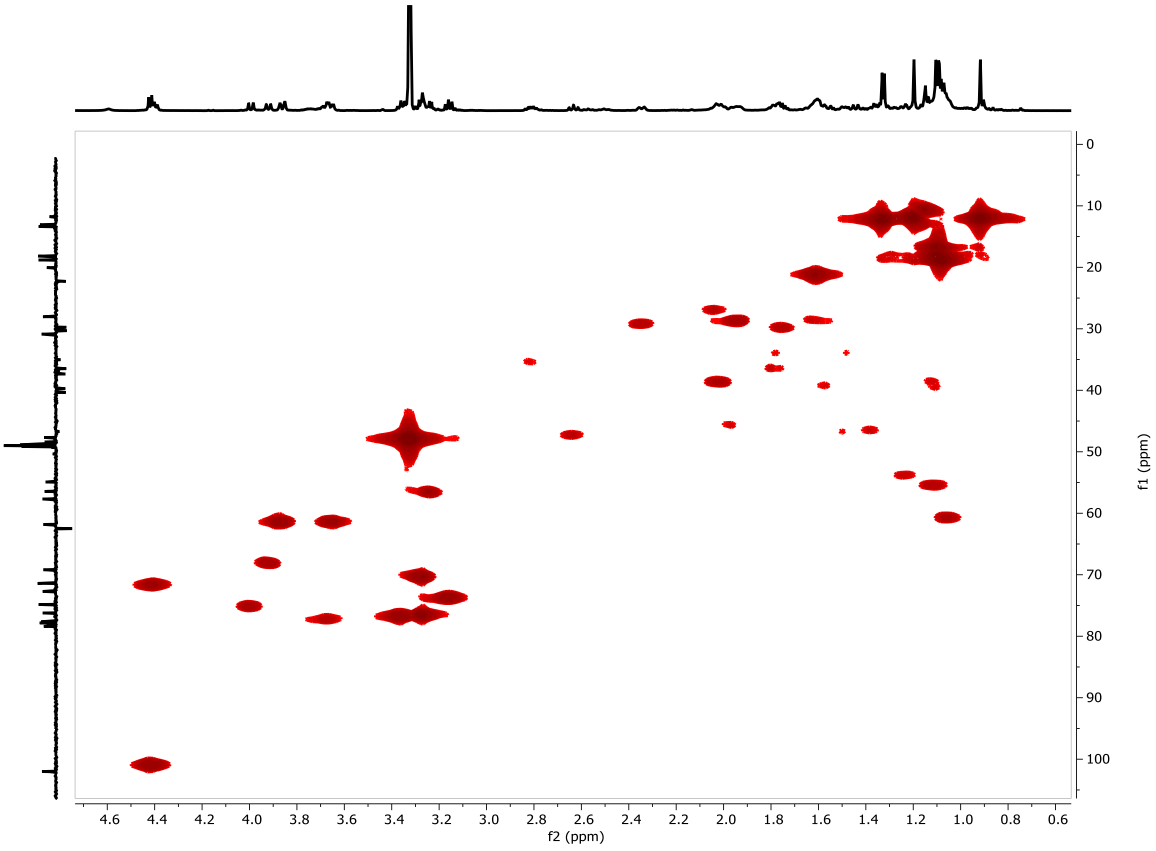

Figure S38: HSQC spectrum of compound **4**

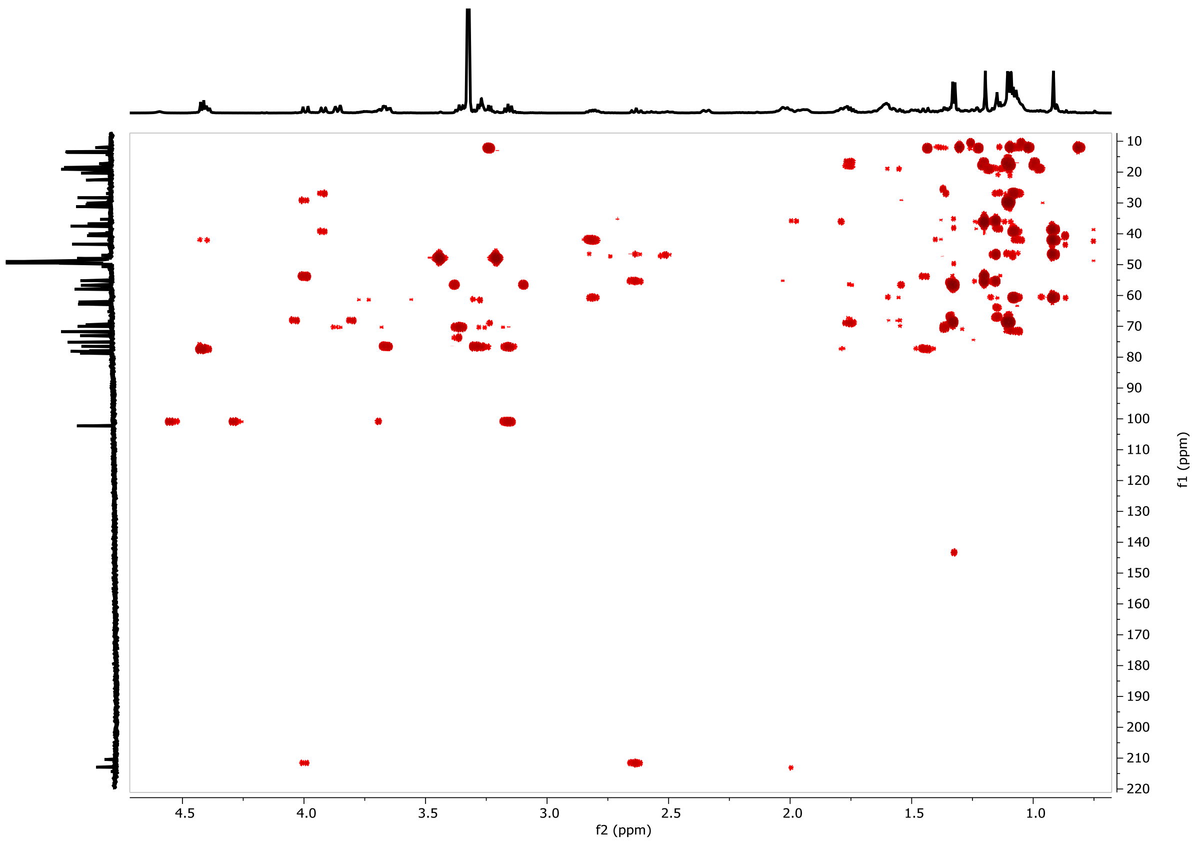

Figure S39: HMBC spectrum of compound **4**

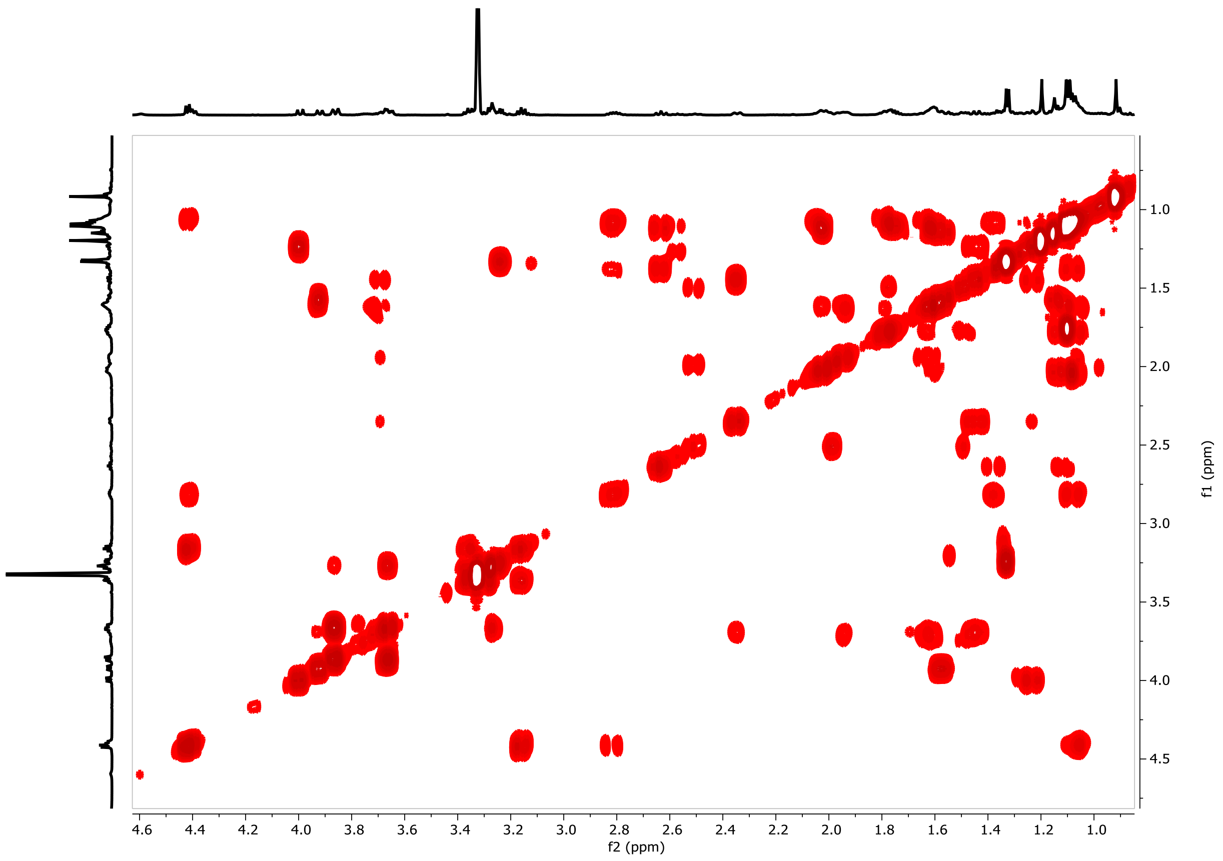

Figure S40: COSY spectrum of compound **4**

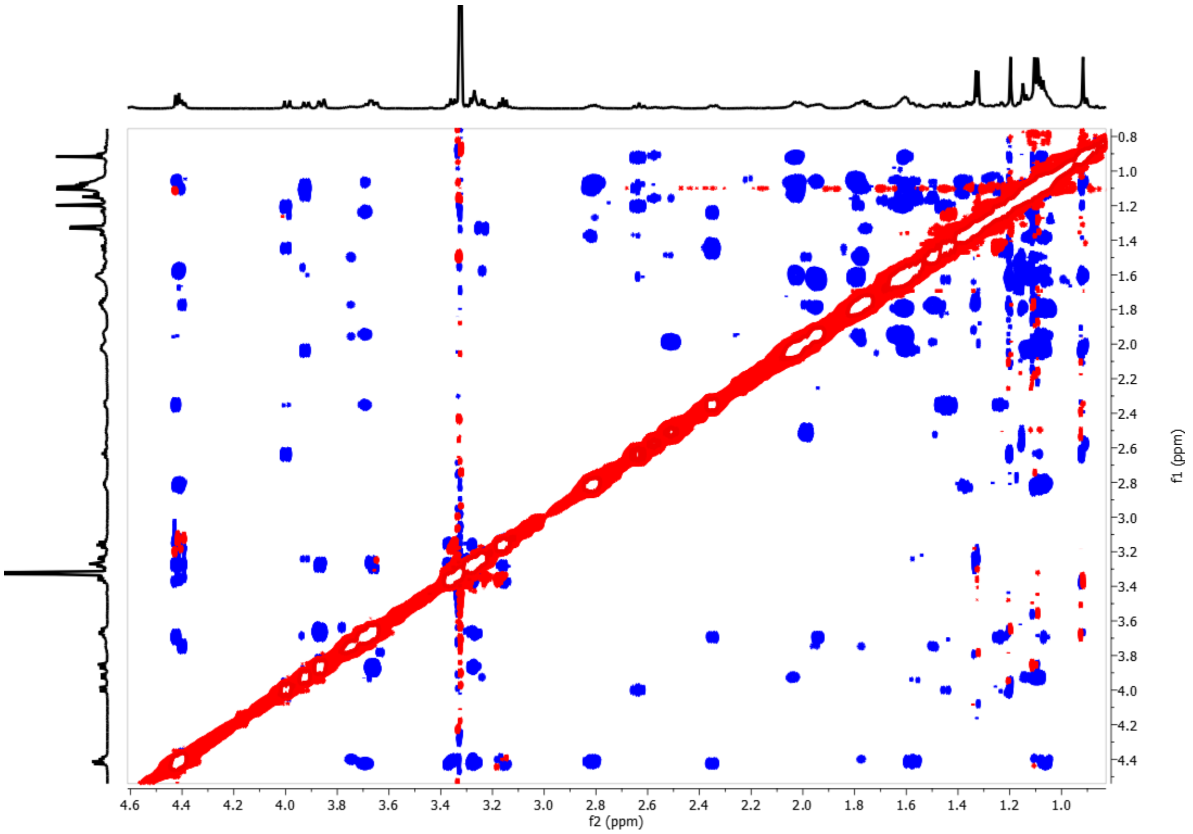

Figure S41: ROESY spectrum of compound **4**

DATA FOR KNOWN COMPOUNDS

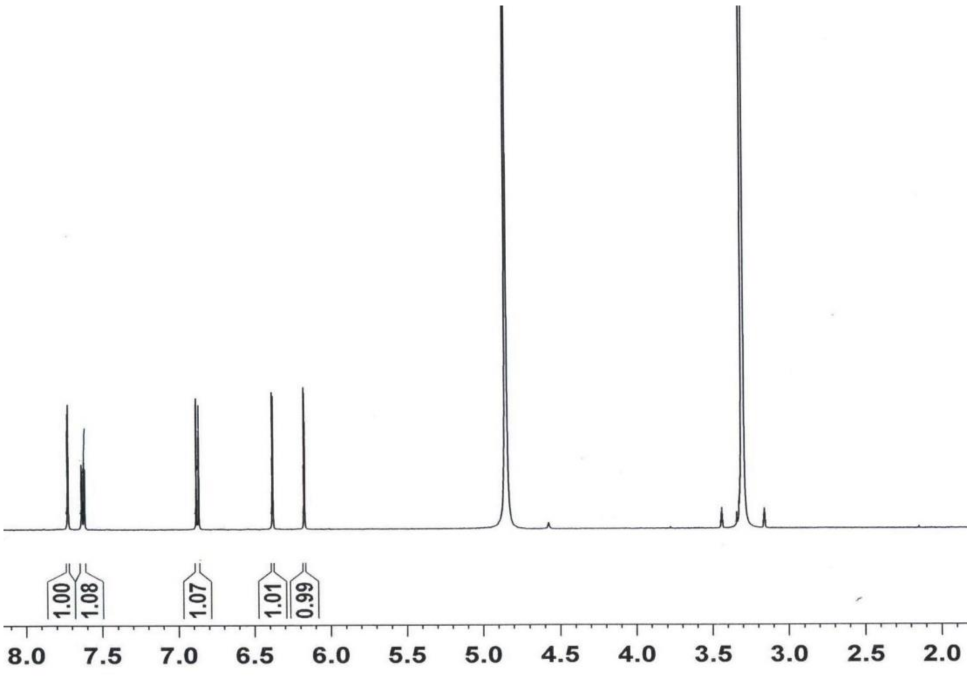

Figure S42: ^{1}H NMR (CD_3OD, 500 MHz) spectrum of compound **5**

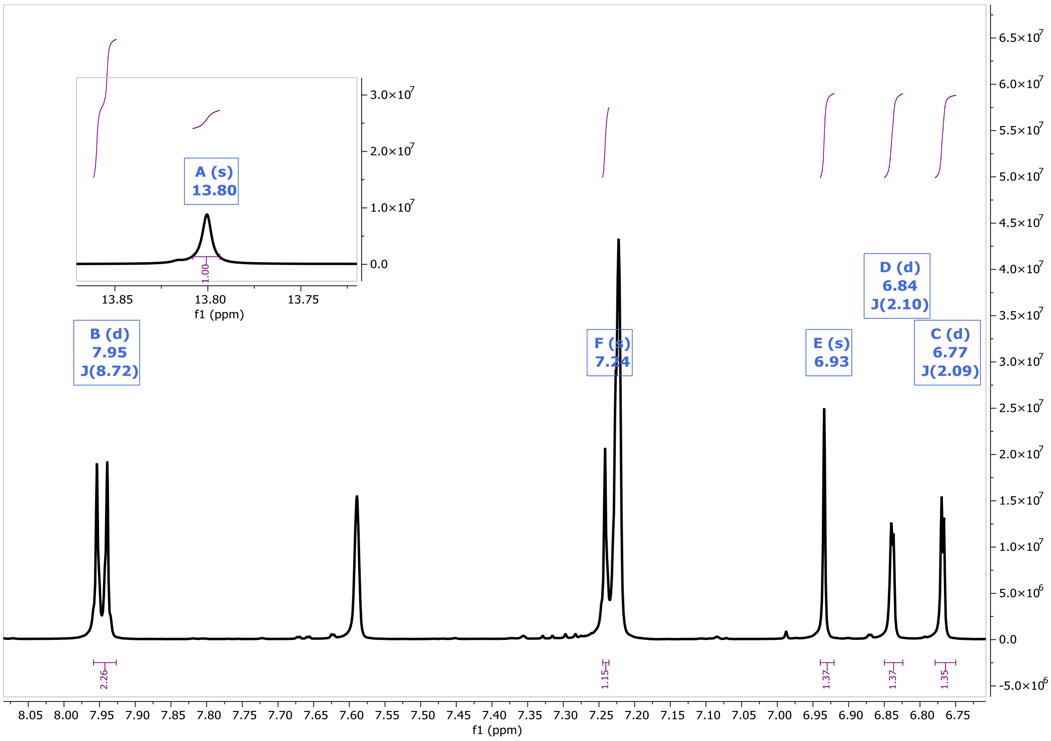

Figure S43: ^{1}H NMR ($CDCl_3$, 500 MHz) spectrum of compound **6**

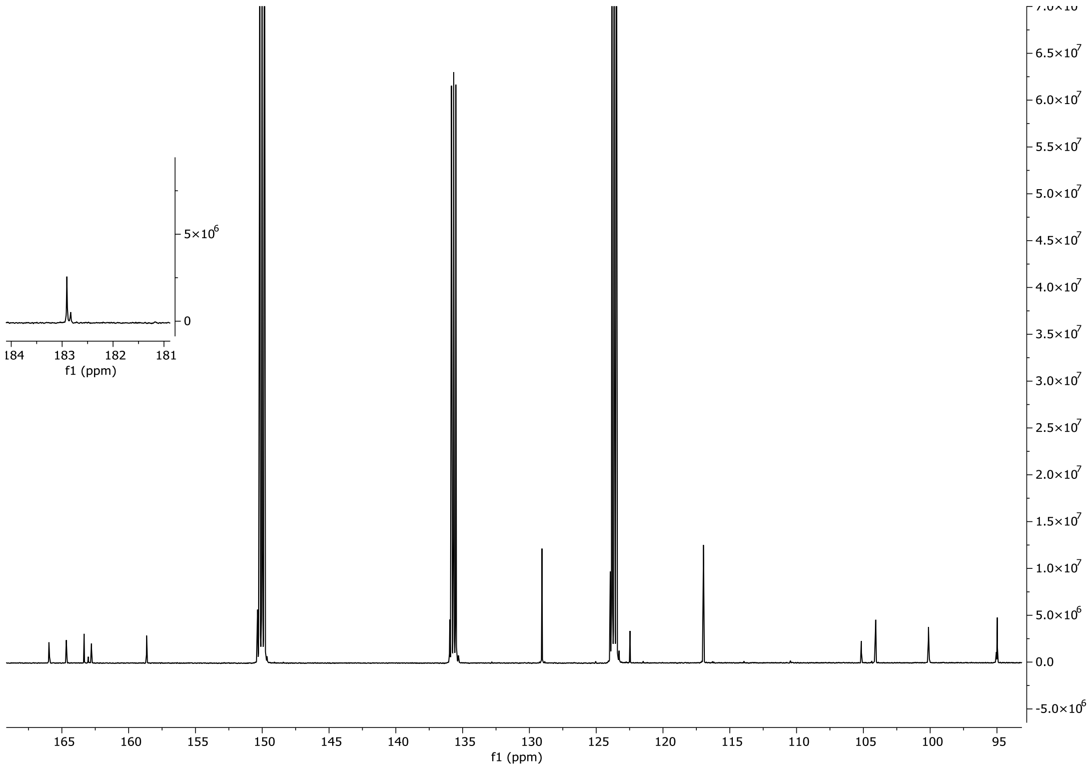

Figure S44: ^{13}C NMR ($CDCl_3$, 125 MHz) spectrum of compound **6**

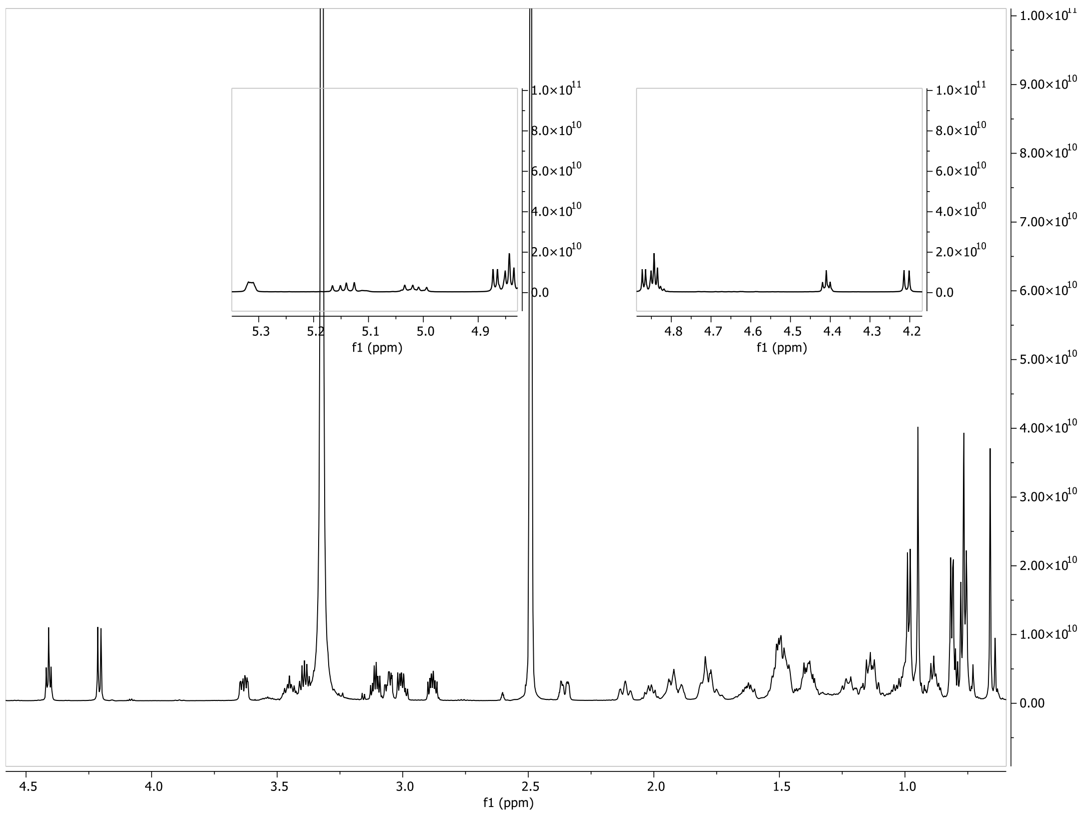

Figure S45: ^{1}H NMR ($CDCl_3$, 500 MHz) spectrum of a mixture of **7** and **8**
